# Overexpression of *OsbHLH107*, a member of the basic helix-loop-helix transcription factor family, enhances grain size in rice (*Oryza sativa* L.)

**DOI:** 10.1186/s12284-018-0237-y

**Published:** 2018-07-20

**Authors:** Xiaoming Yang, Yulong Ren, Yue Cai, Mei Niu, Zhiming Feng, Ruonan Jing, Changling Mou, Xi Liu, Lianjie Xiao, Xin Zhang, Fuqing Wu, Xiuping Guo, Ling Jiang, Jianmin Wan

**Affiliations:** 1 State Key Laboratory for Crop Genetics and Germplasm Enhancement, Research Center of Jiangsu Plant Gene Engineering, Nanjing Agricultural University, Nanjing, 210095 China; 2 National Key Facility for Crop Gene Resources and Genetic Improvement, Institute of Crop Science, Chinese Academy of Agricultural Sciences, Beijing, 100081 China

**Keywords:** Cell cycle, Cell proliferation, bHLH, PILs

## Abstract

**Background:**

Grain size, which is determined by grain length, grain width, and grain thickness, is an important determinant for grain yield in rice. Identification and characterization of new genes that are associated with grain size will be helpful for the improvement of grain yield in rice.

**Results:**

We characterized the grain size mutant, *larger grain size 1* (*lgs1*), derived from rice activation-tagged T-DNA insertion lines. Histological analysis showed that increased cell numbers in the longitudinal direction of spikelet hulls was responsible for the grain mutant phenotype in *lgs1*. Quantitative real-time PCR (qRT-PCR) analysis further showed that the expression levels of genes associated with the cell cycle in the young panicles of the *lgs1* were higher than those in the wild type (WT), which might result in the increased cell numbers in *lgs1* spikelet hulls. Insertion site analysis together with transgenic experiments confirmed that the *lgs1* phenotype was caused by enhanced expression of truncated *OsbHLH107*, corresponding to the nucleotide (nt) 331–846 region (i.e., the transcriptional activation region of OsbHLH107) of the *OsbHLH107* coding sequence (CDS). OsbHLH107 is a nucleus-localized bHLH transcription factor, which can form a homodimer with itself. Phylogenetic analysis showed that OsbHLH107 belonged to the same subfamily as OsPILs. *OsPIL13* (*OsPIL1*) and *OsPIL16* (*APG*) were reported to regulate grain size in rice. By transgenic experiments, we found that *OsPIL11* could also regulate grain size.

**Conclusion:**

We concluded that OsbHLH107 and its homologs are important regulators of grain size development and might be useful for grain yield improvement in rice.

**Electronic supplementary material:**

The online version of this article (10.1186/s12284-018-0237-y) contains supplementary material, which is available to authorized users.

## Background

Rice is an important crop for both human food and research. Genetic and environmental factors determine grain yield. Genetic factors determine potential grain yield, and environmental factors determine how much of that potential can be captured. Almost half a century has passed since the ‘*Green Revolution*’, which was primarily based on semidwarf stature to ensure crop yield potential (Asano et al., 2011).

Grain yield in rice is determined by grain number per panicle, panicle number per plant, and grain weight (Zhou et al., 2017). When grain number per panicle and panicle number per plant reach optimum levels, improvement in grain weight becomes important to further increase grain yield in breeding programs (Jiao et al., 2010; Miura et al., 2010). Grain weight is determined by grain size, which comprises grain length, width, and thickness, which in turn involve cell number and size (Fukada and Kubo, 2015; Jia et al., 2016). *GW2* encodes a RING-type E3 ubiquitin ligase and negatively regulates cell numbers in spikelet hulls (Song et al., 2007); *GW5* is a grain width regulator and acts in the brassinosteroid signaling pathway to regulate grain width by promoting cell division (Shomura et al., 2008; Weng et al., 2008; Duan et al., 2017; Liu et al., 2017); *GW7* and *GW8* have very important roles in grain width regulation, and *GW8* can inhibit the expression of *GW7* to negatively regulate grain size (Wang et al., 2012, 2015a, b). Many genes or QTLs associated with grain length have been reported. *GS3* and *qGL3/qGL3.1* are major QTLs that modulate grain length by controlling cell numbers in the glumes (Fan et al., 2006; Mao et al., 2010; Zhang et al., 2012). *GW6a* encodes a histone H4 acetyltransferase, OsglHAT1. Elevated *GW6a* expression could enhance grain length and weight by increasing cell numbers in spikelet hulls (Song et al., 2015). As a negative regulator of grain length, *TGW6* encodes an IAA-glucose hydrolase protein. Loss of function of *TGW6* will lift restrictions on IAA supply, resulting in increased cell numbers in spikelet hulls (Ishimaru et al., 2013). *SRS3* encodes a protein that contains a kinesin motor domain and a coiled-coil structure. The grain length of *srs3* is shorter than that of WT, due to a reduction in the cell length of spikelet hulls in the longitudinal direction (Kitagawa et al., 2010; Wu et al., 2014). *SRS5* encodes an alpha tubulin protein and has a similar role as *SRS3* in regulation of grain length (Segami et al., 2012).

The basic helix-loop-helix (bHLH) proteins are a group of transcription factors that play various roles in plant development, and 167 bHLH proteins have been identified in the rice genome (Li et al., 2006). The bHLH motif contains two functionally distinctive regions: the basic (b) region for DNA-binding and the helix-loop-helix (HLH) region for protein dimerization (Massari and Murre, 2000). Based on DNA-binding ability, proteins that can bind DNA are called DNA-binding bHLH (typical bHLH), and the others are called non-DNA-binding bHLH (HLH or atypical bHLH) (Li et al., 2006). Recent studies have revealed that atypical bHLH proteins undergo heterodimerization with typical bHLH proteins through the bHLH domain and function antagonistically (Toledo-Ortiz, 2003). For example, *Increased Leaf Inclination* (*ILL1*) and *ILI1 binding bHLH* (*OsIBH1*) act antagonistically to control cell length in the lamina joint (Zhang et al., 2010). PIFs/PILs (Phytochrome-Interacting Factors/Phytochrome-Interacting factors-Like) are a subgroup within the bHLH transcription factor family. In Arabidopsis, PIFs interact with PHYs (Phytochromes) to regulate perception of light signals and affect heading (Leivar and Monte, 2014; Luo et al., 2014; Sakuraba et al., 2014; Soy et al., 2014). Six OsPILs (OsPIL11-OsPIL16) were identified by searching rice genomic database. Each of them encodes a protein with a PIL motif that is highly homologous to PIFs in *Arabidopsis thaliana* (Nakamura et al., 2007). Whether these OsPILs can interact with OsPHYs to regulate perception of light signals and induce heading in rice has not been determined. OsPIL13 (also named OsPIL1) and OsPIL16 (also named APG), but not OsPIL15*,* are regulators of grain length, but the functions of OsPIL11, OsPIL12, and OsPIL14 have not been reported. OsPIL13 is a positive regulator of grain length (Todaka et al., 2012). Three *OsbHLH* genes with antagonistic effects on grain size were identified: *PGL1*, *PGL2*, and *APG* (*OsPIL16*). PGL1 and PGL2 are positive grain length regulators, and APG belongs to the OsPIL family, which has functionally antagonistic effects on PGL1 and PGL2 as a negative grain length regulator (Heang and Sassa, 2012a, b, c).

In this study, we isolated and characterized a rice grain size mutant, *lgs1*, which has enhanced expression of *OsbHLH107*, a member of the bHLH transcription factor family that regulates grain size by influencing cell numbers in the longitudinal direction of spikelet hulls. We also found that OsPIL11, a homolog of OsbHLH107, regulates grain size.

## Results

### Characterization of *lgs1*

**Fig. 1:**
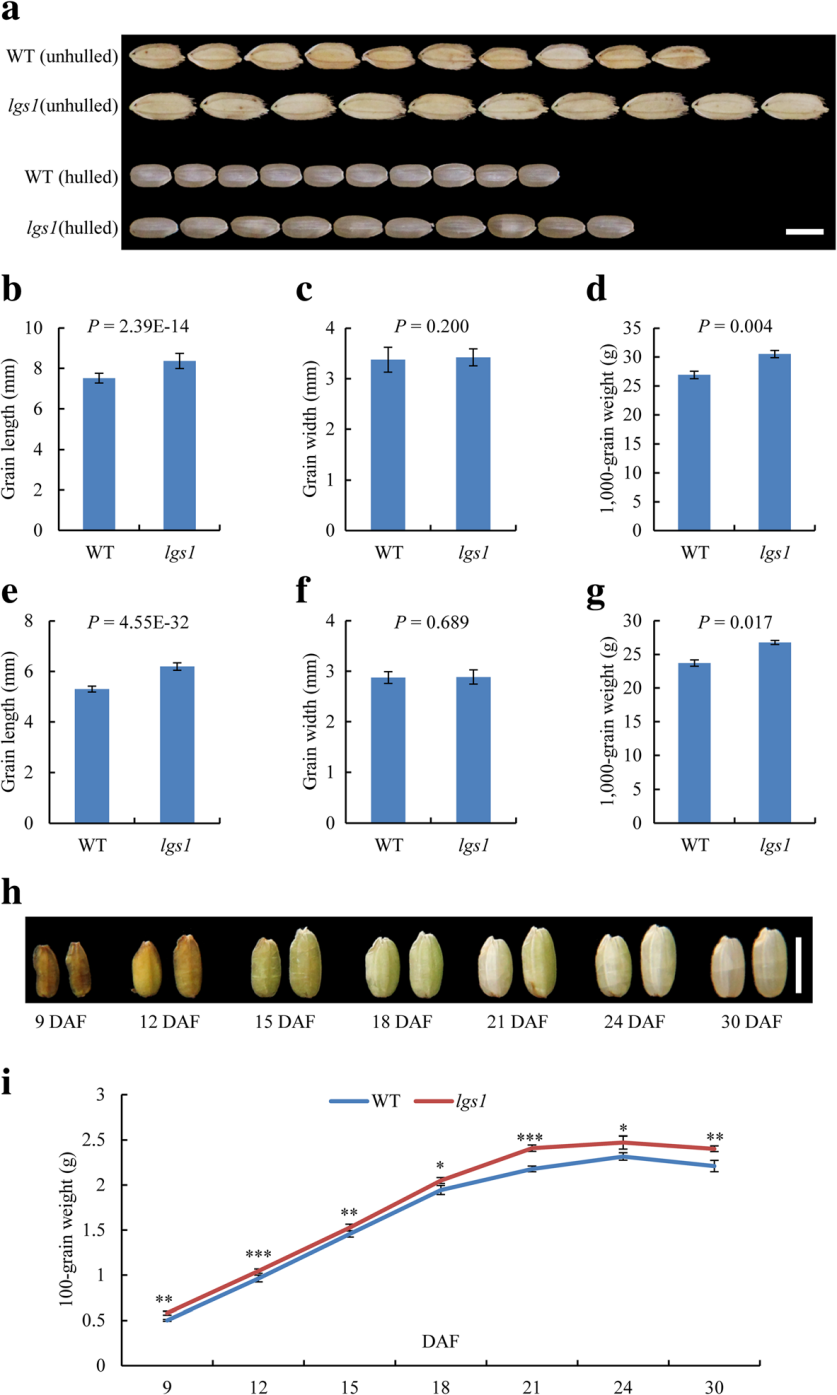
Comparisons of grain size between ‘Dongjin’ (WT) and *lgs1*. **a** Comparison of WT and *lgs1* seeds (unhulled and hulled). Scale bar, 5 mm. **b-d** Comparisons of grain length (**b**, *n* = 30), grain width (**c**, *n* = 30), and 1000-grain weight (**d**, *n* = 3) of unhulled seeds between WT and *lgs1*. **e-g** Comparisons of grain length (**e**, *n* = 30), grain width (**f**, *n* = 30), and 1000-grain weight (**g**, *n* = 3) of hulled seeds between WT and *lgs1*. **h** Grain appearance of WT (left) and *lgs1* (right) at a series of developmental stages as indicated. Scale bar, 5 mm. **i** Grain-filling process of WT and *lgs1* (*n* = 5). Data are the mean ± SD. Student’s *t*-tests were used to generate the *P* values. *P ≤ 0.05, **P ≤ 0.01, ***P ≤ 0.001

As part of our continuing efforts to investigate the molecular mechanisms regulating grain size, we searched for mutants with altered grain size from a collection of activation-tagged T-DNA insertion lines (Jeong et al., 2002, 2006). One mutant, named *lgs1* on a *japonica* cultivar (cv.) ‘Dongjin’ (WT) background, displayed increased grain size compared to WT. At the heading stage, *lgs1* showed almost the same plant architecture as WT (Additional file 1: Figure S1a-c). Statistical analysis showed no significant differences in plant height, heading date, tiller number per plant, primary branch number per panicle, spikelet number per panicle, and fully filled grain number per panicle between WT and *lgs1* (Additional file 1: Figure S1d-i). However, grain size, especially grain length, of *lgs1* appeared larger than that of WT (Fig. 1a). Statistical analysis showed that grain length and 1000-grain weight were significantly increased in *lgs1* (Fig. 1b, d, e and g), but grain width was not significantly changed (Fig. 1c and f). To characterize the *lgs1* phenotype in detail, we performed time-course analysis of grain development from 9 days after fertilization (DAF) to 30 DAF. Notably, *lgs1* developed larger grains than WT early at 9 DAF (Fig. 1h and i).

### Cell number increased in *lgs1* spikelet hulls

**Fig. 2:**
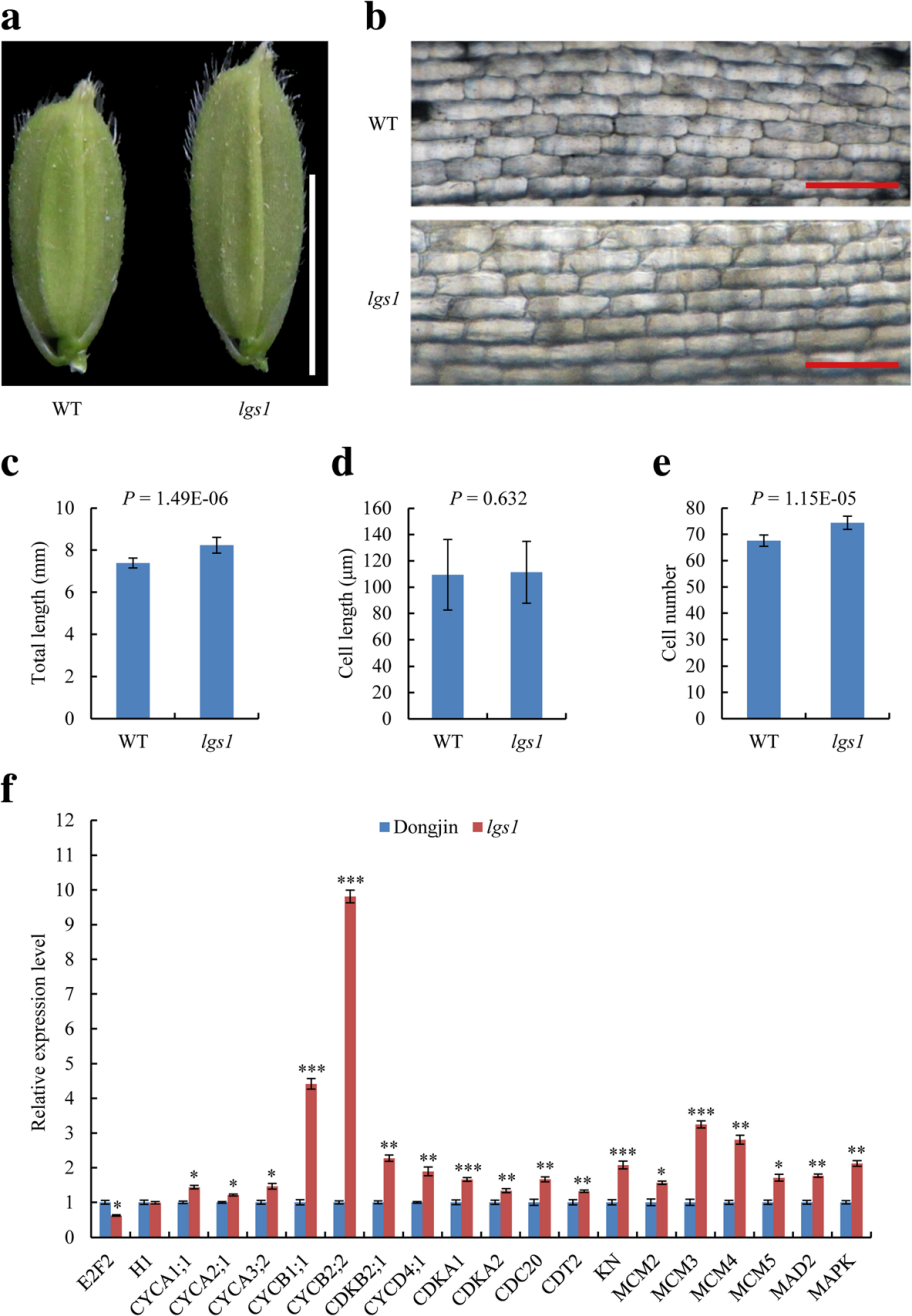
Increased cell number of spikelet hulls in the longitudinal direction determines *lgs1* phenotype. **a** Young spikelet hulls of ‘Dongjin’ (WT) and *lgs1*. Scale bar, 5 mm. **b** Electron microscopic observation of inner epidermal cells. Scale bar, 100 μm. **c-e** Total length (**c**, *n* = 10), cell length (**d**, *n* = 100), and cell number (**e**, *n* = 10) of inner epidermal cells. **f** Relative expression levels of genes associated with the cell cycle. Relative expression levels of each gene were determined by qRT-PCR with RNA isolated from 5 cm panicles of WT and *lgs1*. *OsActin1* was used as the internal control, and the values of the relative expression levels in the WT were set to one (*n* = 3). Data are the mean ± SD. Student’s *t*-tests were used to generate the *P* values. *P ≤ 0.05, **P ≤ 0.01, ***P ≤ 0.001

To further clarify the causes of the longer grain in *lgs1*, we examined both cell size and cell number of spikelet hulls of WT and *lgs1*. Histological observations and statistical analysis showed that the average length of the inner epidermal cells of spikelet hulls in the longitudinal direction was not significantly changed in *lgs1* compared with WT (Fig. 2a-d), while the cell number was significantly increased (Fig. 2e). Consistent with this finding, the relative expression levels of many genes involved in the cell cycle pathway were considerably higher in *lgs1* than the WT (Fig. 2f, Additional file 1: Table S2), suggesting that the increased cell numbers in spikelet hulls of *lgs1* might be due to increased expression levels of genes known to promote cell proliferation. These results indicated that *lgs1* controls grain size most likely by influencing cell numbers in the longitudinal direction of spikelet hulls.

### Enhanced expression of *OsbHLH107* caused a large grain phenotype

**Fig. 3:**
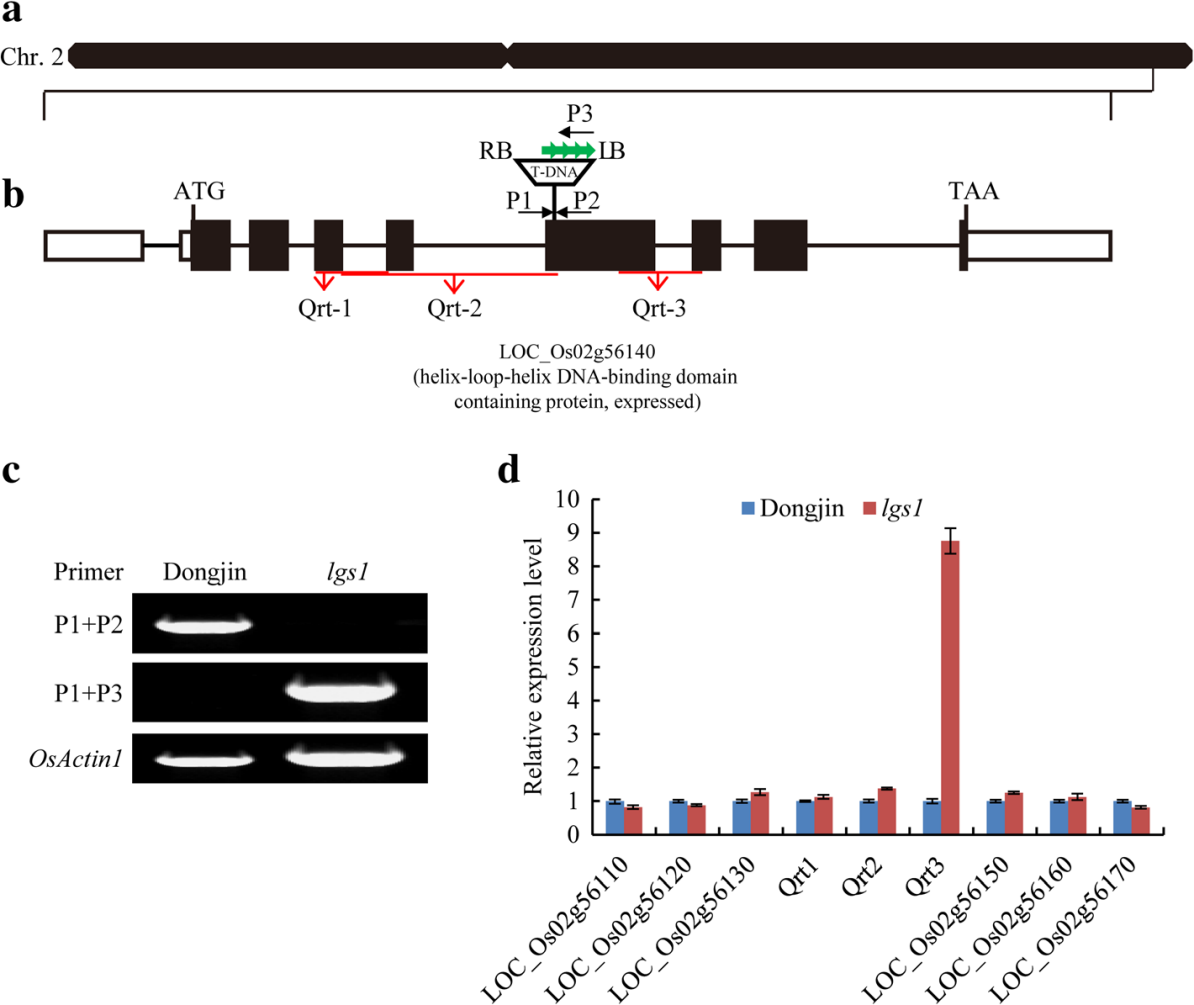
Identification of the T-DNA insertion site in *lgs1*. **a** *OsbHLH107* (*LOC_Os02g56140*) located at the end of rice chromosome 2. Black lines, black rectangles, and white rectangles in the genomic region represent introns, exons, and UTRs, respectively. **b** The T-DNA insertion site in *lgs1*. ATG and TAA represent the start and stop codons in the CDS, respectively. The four copies of 35S enhancers in the T-DNA insertion sequence are indicated with green arrows. RB and LB indicate the T-DNA vector right and left borders, respectively. P1, P2, and P3 are primers used for identifying the T-DNA insertion site. Qrt1, Qrt2, and Qrt3 are primer pairs used for qRT-PCR analysis of different regions of *LOC_Os02g56140*. **c** Identification of the T-DNA insertion by PCR analysis. **d** qRT-PCR analysis of different regions of *LOC_Os02g56140* and the genes upstream or downstream of it, with RNA isolated from leaves. *OsActin1* was used as the internal control, and the relative expression levels in the WT were set to one (*n* = 3). Data are the mean ± SD

To isolate the gene responsible for the *lgs1* (line 1B-04807) phenotype, we obtained the genomic flanking sequence from the T-DNA insertion database OryGenesDB (http://orygenesdb.cirad.fr/). pGA2715, an activation-tagged T-DNA vector with four copies of 35S enhancers on the left border (LB), was integrated into the 5th exon of *LOC_Os02g56140*, which encodes OsbHLH107, a member of the bHLH transcription factor family (Fig. 3b). PCR analysis further confirmed the T-DNA insertion site in *lgs1* (Fig. 3c). A BLAST search (http://rice.plantbiology.msu.edu/) showed that *OsbHLH107* is located at the end of rice chromosome 2 (Fig. 3a), with the genes *LOC_Os02g56110*, *LOC_Os02g56120*, and *LOC_Os02g56130* upstream and *LOC_Os02g56150*, *LOC_Os02g56160*, and *LOC_Os02g56170* downstream. To determine how the transcription of *LOC_Os02g56140* was affected by the T-DNA insertion, we performed a 5′ rapid amplification of cDNA ends (5’ RACE) experiment. The 5’ RACE experiment showed that transcription of the region after the T-DNA insertion site was not disrupted, and at least two truncated transcripts were obtained (Additional file 1: Figure S2). Nine primer pairs were designed to examine whether the four 35S enhancers in the pGA2715 T-DNA vector had an influence on the expression levels of different regions of the *LOC_Os02g56140* mRNA, and the genes upstream or downstream of it. qRT-PCR analysis showed that only the region after the T-DNA insertion site (primer pair, Qrt3) exhibited higher expression levels in *lgs1* than WT (Fig. 3d). These results indicated that elevated expression of the truncated *OsbHLH107* transcripts in *lgs1* might be responsible for the mutant phenotype.

**Fig. 4:**
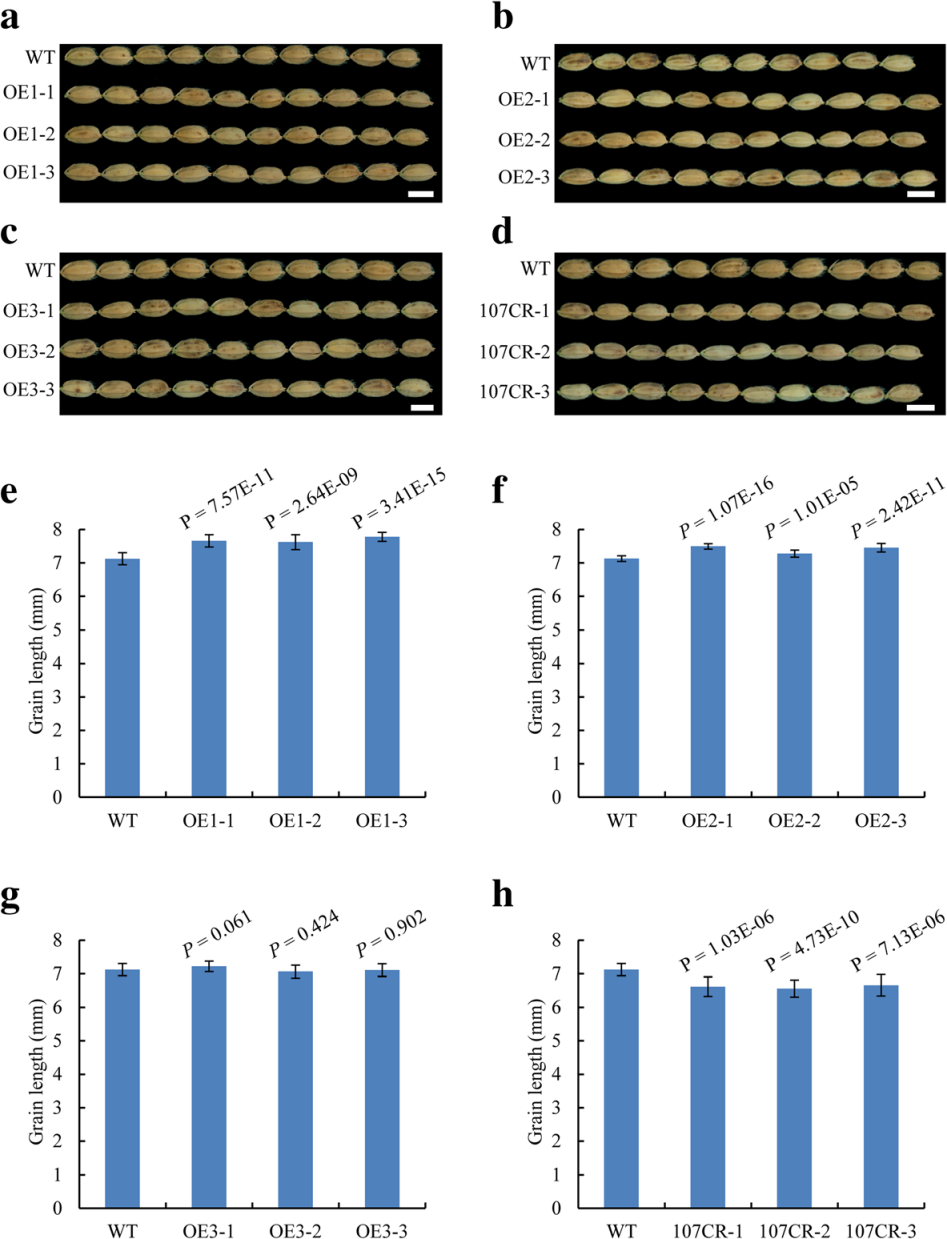
*OsbHLH107* acts as a positive regulator in controlling grain development. **a-d** Grain length appearance of OE1 (**a**), OE2 (**b**), OE3 (**c**), and *OsbHLH107*-CRISPR/Cas9 (**d**, 107CR) transgenic plants on a ‘Dongjin’ (WT) background. Scale bar, 5 mm. **e**-**h** Statistical analysis of grain length shown in (**a-d)**, respectively (*n* = 20). Data are the mean ± SD. Student’s *t*-tests were used to generate the *P* values

The *OsbHLH107* gene was predicted to encode a protein composed of 281 amino acid (aa) residues that harbors a putative bHLH domain in the N-terminal and middle region (aa 51–110, Additional file 1: Figure S3). To test our assumption that overexpression of the truncated *OsbHLH107* transcripts in *lgs1* might be responsible for the mutant phenotype, we overexpressed (OE) nt 331–846 (i.e., aa 111–281) region, which contains the full-length sequence of altered transcripts after the T-DNA insertion site, under the control of the maize *Ubi* promoter (*pUbi::*CDS^*OsbHLH107* (nt 331–846)^, OE1) on a WT background. Statistical analysis showed that grain length of the transgenic plants was significantly increased compared to that of the WT (Fig. 4a and e; Additional file 1: Figure S4a). We also overexpressed full-length CDS (*pUbi::*CDS^*OsbHLH107* (nt 1–846)^, OE2) and nt 1–330 (*pUbi::*CDS^*OsbHLH107* (nt 1–330)^, OE3) of *OsbHLH107*. Interestingly, only transgenic plants overexpressing the full-length *OsbHLH107* gene displayed significantly increased grain length (Fig. 4b, c, f and g; Additional file 1: Figure S4b and c). These results suggested that *OsbHLH107* might positively regulate grain development in rice. To further confirm this, we generated *OsbHLH107* knock-out and knock-down transgenic plants using CRISPR/Cas9 (CR) and RNA interference (RNAi) technologies. As expected, loss of function of *OsbHLH107* on a WT background substantially reduced the grain length (Fig. 4d and h; Additional file 1: Figure S4d and Figure S12), and the same results were also obtained through RNAi experiments (Additional file 1: Figure S5). Collectively, these data suggested that the T-DNA insertion in *lgs1* is responsible for the elevated expression of nucleotides after the T-DNA insertion site, which enhances grain size.

### *OsbHLH107* encodes a nucleus-localized protein that can form homodimers

**Fig. 5:**
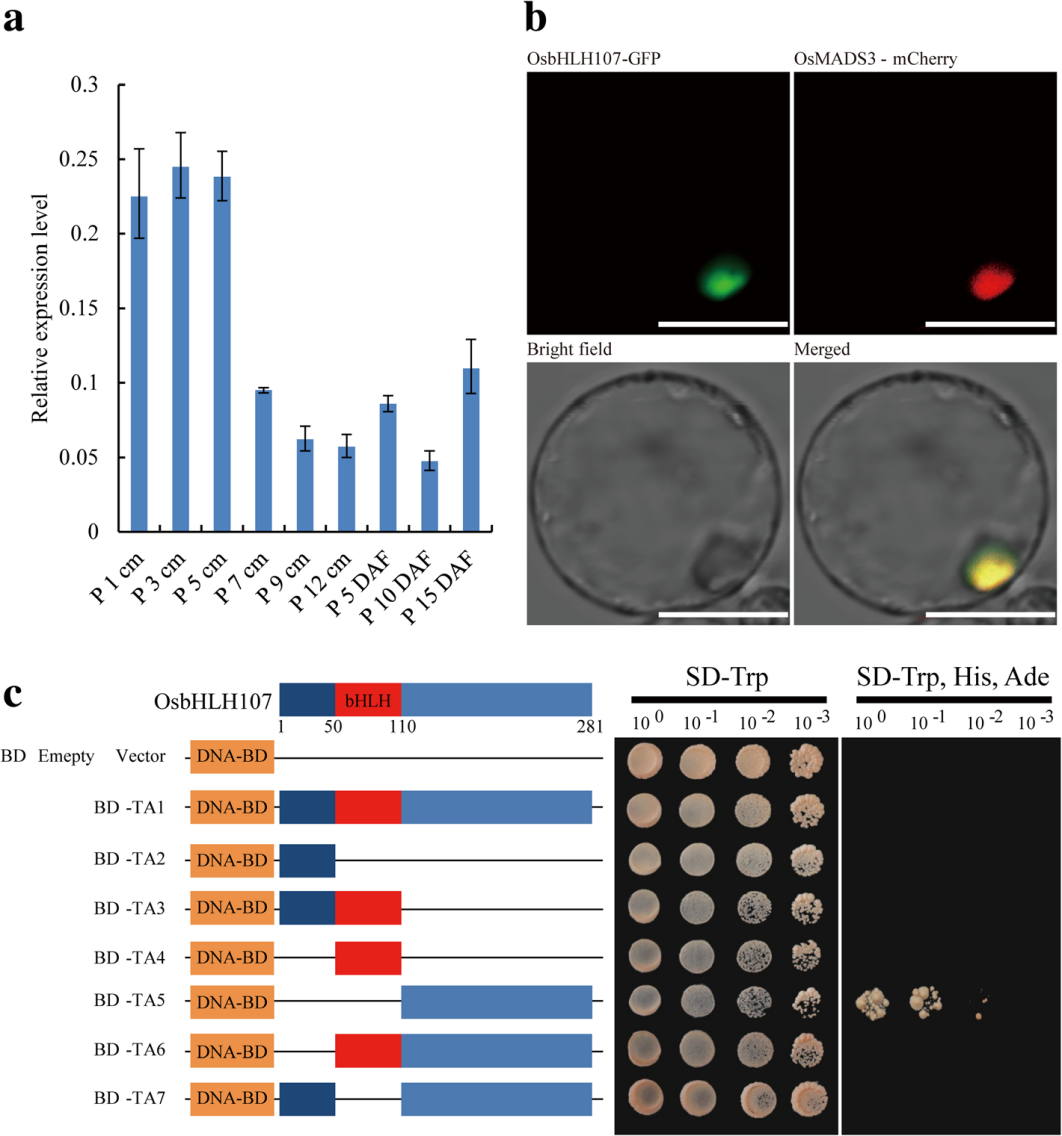
Expression patterns, subcellular-localization, and transcriptional activity analysis of *OsbHLH107*. **a** Relative expression levels of *OsbHLH107* in various developmental stages of the panicles. P, panicles. *OsActin1* was used as the internal control (*n* = 3). Data are the mean ± SD. **b** The OsbHLH107-GFP fusion protein was located in the nucleus in rice protoplasts. Scale bar, 20 μm. **c** Transcriptional activity assays of OsbHLH107 and its series of domain deletion variants in the yeast GAL4 system. Numbers below the schematic represent amino acid positions of OsbHLH107*.* DNA-BD represents the GAL4 DNA-binding domain. Empty pGBKT7 vector was used as the negative control

**Fig. 6:**
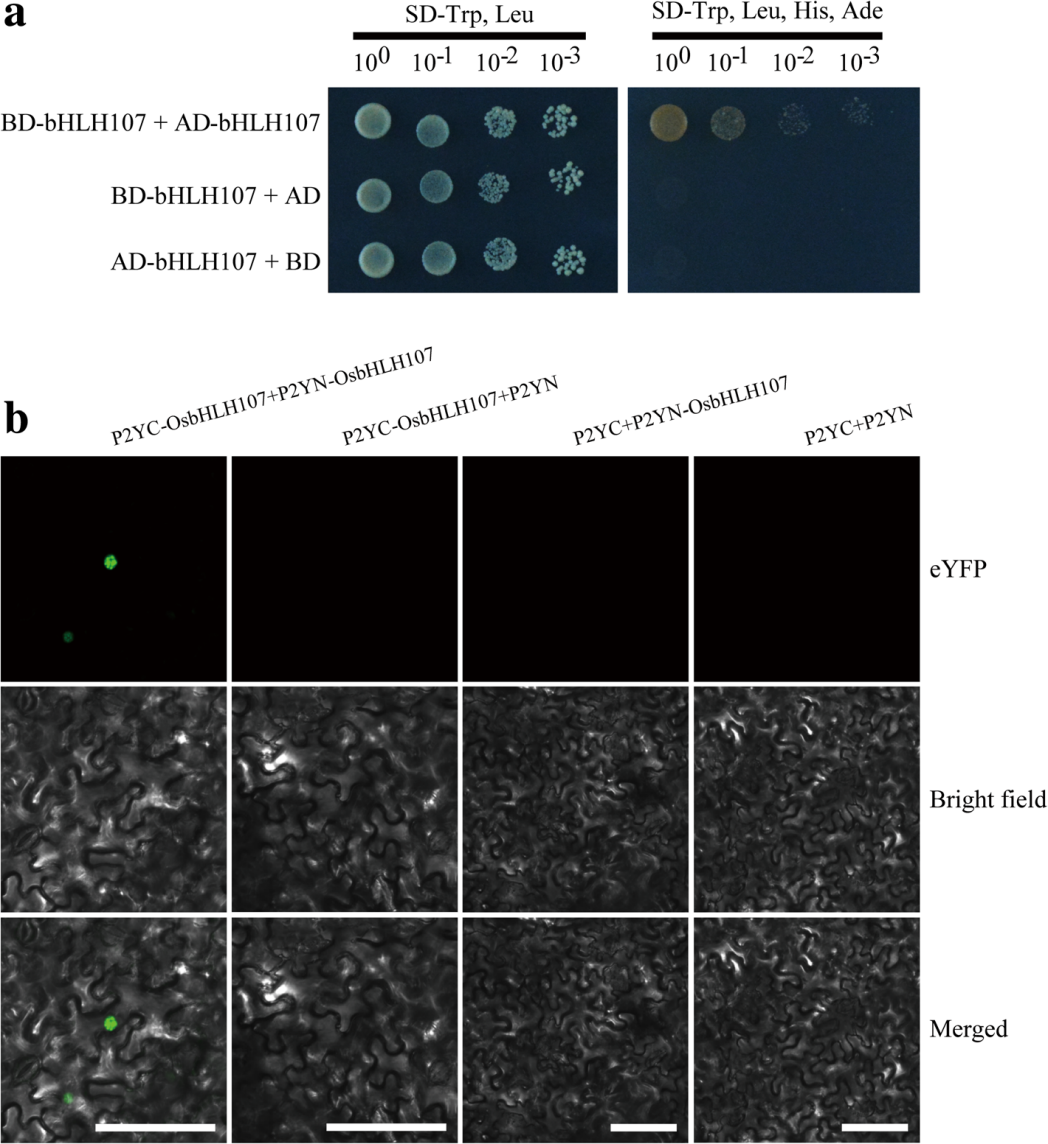
OsbHLH107 protein can form homodimers in vitro and in vivo. **a** Y2H assays showing that OsbHLH107 physically interacts with itself. AD, active domain. BD, binding domain. **b** BiFC assay showing that OsbHLH107 interacts with itself in leaf cells of *Nicotiana benthamiana*. YFP, yellow fluorescent protein. Scale bar, 100 μm

A search of the Rice Expression Profile Database (http://ricexpro.dna.affrc.go.jp/) showed that *OsbHLH107* is expressed throughout plant development (Additional file 1: Figure S6). To further examine the expression profile of *OsbHLH107* at various developmental stages of the panicle in more detail, we performed qRT-PCR analysis and found that *OsbHLH107* showed the highest expression in young panicles of 1 cm, 3 cm, and 5 cm in length, and then, the expression level gradually declined (Fig. 5a). Together, these data suggested that *OsbHLH107* is broadly expressed in various tissues, with the highest expression in the early stages of panicle development. bHLH domain-containing proteins represent a group of transcription factors that putatively function in the nucleus (Li et al., 2006). To verify the subcellular-localization pattern of OsbHLH107, we transiently expressed the *p35S::OsbHLH107-GFP* vector in rice protoplasts. As expected, the OsbHLH107-GFP fusion protein was exclusively co-localized with the nucleus marker (OsMADS3-mCherry) (Li et al., 2011), indicating that OsbHLH107 indeed functions in the nucleus (Fig. 5b). We further fused full-length and various domain deletion variants of OsbHLH107 with the GAL4 DNA-binding domain to map its transcriptional activation region in yeast. As shown in Fig. 5c, only the C-terminal 171 amino acid residues could activate the reporter gene expression. This observation suggested that the activation domain is located in the C-terminus of OsbHLH107, which corresponds to the region responsible for the mutant phenotype (Fig. 4). Generally, bHLH transcription factors form protein dimerization (Massari and Murre, 2000; Li et al., 2006). A yeast two-hybrid (Y2H) assay showed that OsbHLH107 indeed interacted with itself (Fig. 6a). In addition, a bimolecular fluorescence complementation (BiFC) experiment confirmed the in vivo interaction of OsbHLH107 with itself in the nucleus of leaf epidermal cells of *Nicotiana benthamiana* (Fig. 6b). We also found that both aa 1–110 and aa 111–281, the truncated forms of the OsbHLH107 protein, were able to localize in the nucleus (Additional file 1: Figure S7) and had the ability to form homodimers (Additional file 1: Figure S8).

### Phylogenetic analysis of OsbHLH107 and function analysis of OsPIL11

**Fig. 7:**
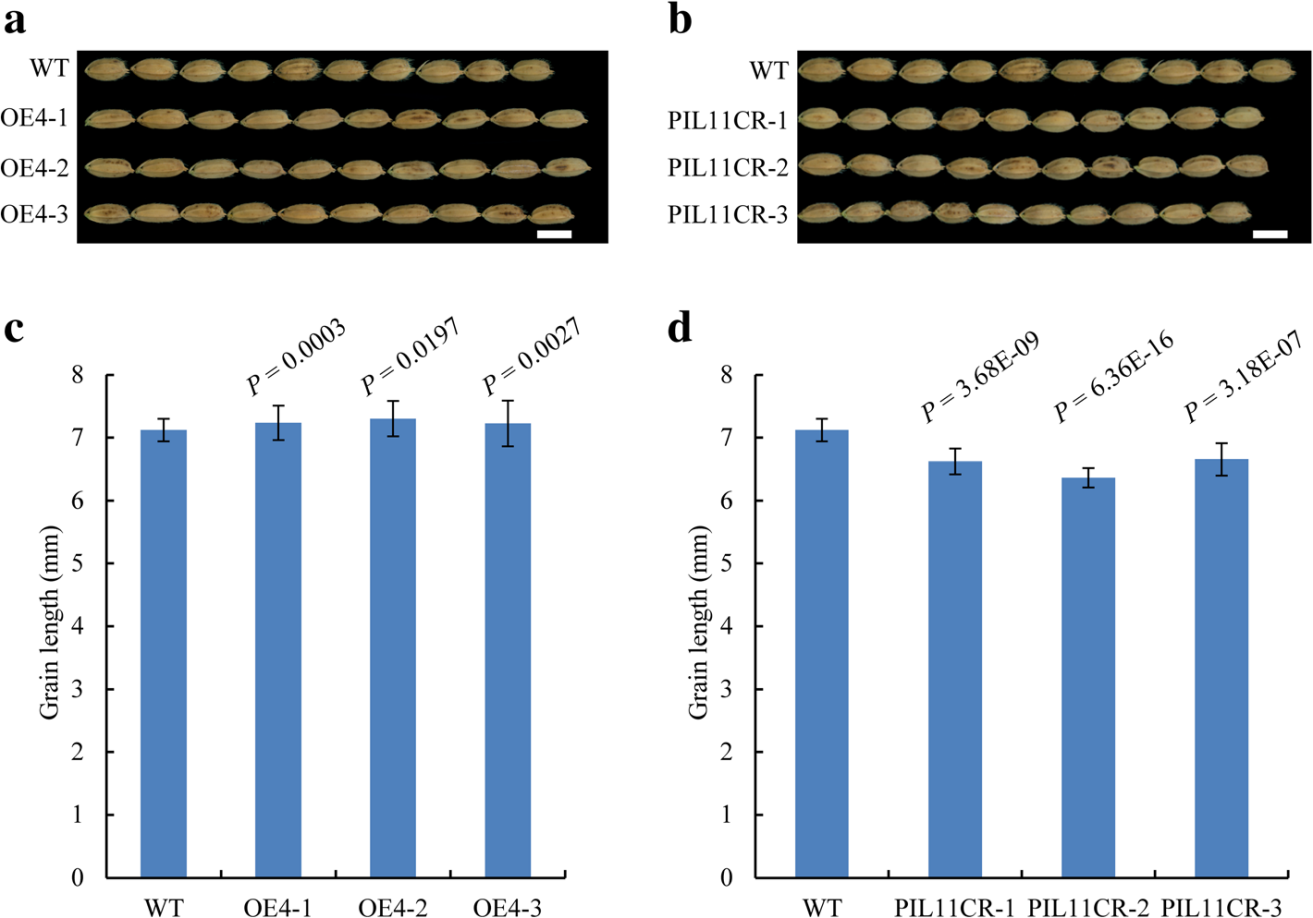
*OsPIL11* is also a grain size regulator. Grain length appearance of *pUbi::*CDS^*OsPIL11*^ (**a**, OE4) and *OsPIL11*-CRISPR/Cas9 (**b**, PIL11CR) transgenic plants on a ‘Dongjin’ (WT) background. Scale bar, 5 mm. **c** and **d** Statistical analysis of grain length shown in (**a** and **b)**, respectively (*n* = 20). Data are the mean ± SD. Student’s *t*-tests were used to generate the *P* values

OsbHLH107 was reported to belong to the same phylogenetic subfamily as OsPILs (Li et al., 2006). We constructed a bHLH family phylogenetic analysis using 27 reported OsbHLH family proteins (Additional file 1: Table S3, China Rice Data Center, http://www.ricedata.cn/).The phylogenetic analysis showed that the amino acid sequences of OsbHLH107 and OsPILs are in highly similarity (Additional file 1: Figure S9). A BLAST search showed that OsbHLH107 harbors only one bHLH domain in the N-terminal and middle region (aa 51–110; Additional file 1: Figure S3). Therefore, OsbHLH107 might not belong to the OsPILs family due to the lack of the PIL motif. OsPIL13 (OsPIL1) and OsPIL16 (APG), but not OsPIL15, are known regulators of grain size, but the functions of OsPIL11, OsPIL12, and OsPIL14 have not been reported (Todaka et al., 2012; Heang and Sassa, 2012a, b). To clarify the functions of OsPIL11 and OsPIL14 (we could not obtain the transcript of *OsPIL12*), we overexpressed their full-length CDSs, and those of knock-out mutants were also obtained by the CRISPR/Cas9 technology. Overexpression of *OsPIL11* significantly increased grain length, whereas the *OsPIL11*-CRISPR/Cas9 knock-out plants had short grains (Fig. 7; Additional file 1: Figure S10 and Figure S12), similar to the transgenic experiment results of *OsbHLH107*. These results indicated that OsbHLH107 and its homologs may play roles in regulation of grain size.

## Discussion

In this study, we proved that *OsbHLH107* regulates grain size by influencing cell numbers in the longitudinal direction of spikelet hulls. Transgenic experiments confirmed that overexpression of either the full-length or the nt 331–846 region of the *OsbHLH107* CDS could enhance grain size, which are similar to *lgs1*. On the other hand, knock-out (CRISPR/Cas9) or knock-down (RNAi) of *OsbHLH107* reduced the grain length. However, we found that regardless of whether the full-length *OsbHLH107* CDS or the nt 331–846 region was used, the effect on grain length did not reach the level of *lgs1*. The present study only simulated the mutation at the transcriptional level, and how these changes in the transcript sequence after the T-DNA insertion site affect gene function at the protein level requires further study. Moreover, we could not rule out the possibility that some other mutations induced by tissue culture also partly conferred to the enlarged grain size.

OsbHLH107 is a typical bHLH (with a DNA-binding element known as a G-box). In theory, because of the lack of the bHLH domain, this truncated protein (aa 111–281) of OsbHLH107 may not bind DNA and interact with its partners. However, through our experiments, we found that this truncated protein is also located in the nucleus and can form homodimers. These results indicated that this truncated protein may interact with other proteins in the nucleus to regulate the expression of downstream genes. Unfortunately, we have not yet identified proteins that can interact with OsbHLH107 to prove our hypothesis; this will be the focus of future research on the function of OsbHLH107. In addition, although aa 1–110 of OsbHLH107 was also located in the nucleus and could form homodimers, it did not have the ability to regulate grain size directly, possibly because of a lack of the transcriptional activation ability. This further indicated that the transcriptional activation ability of aa 111–281 was important for the OsbHLH107 function.

Phylogenetic analysis showed that OsbHLH107 has a tight evolutionary linkage with OsPILs. To investigate whether there are functional correlations between OsbHLH107 and its homologous genes, we conducted qRT-PCR analysis and found that there were no obvious differences at the expression levels between WT and *lgs1* (Additional file 1: Figure S11). Transgenic experiments showed that overexpression of *OsPIL11* significantly increased grain length and *OsPIL11*-CRISPR/Cas9 knock-out plants had short grains. In addition, although nucleotide changes were in 5’ UTR region of *OsPIL11*-CRISPR/Cas9 mutants, we think that these changes may affect the stability and/or expression of *OsPIL11* mRNA, which leads to the mutant phenotype, and similar effects of UTR changes on mRNA stability and/or expression have been reported in several previous studies (Hughes, 2006; Srivastava et al., 2017). All these results indicated that OsbHLH107 and its homologs (OsPIL1/13, OsPIL11, OsPIL16) play roles in regulation of grain size but may act independently.

Plant organ size is determined by cell division and cell expansion (Sugimoto-Shirasu and Roberts, 2003). Many *OsbHLH* genes participate in regulation of plant organ size. For example, *An-1* encodes a bHLH protein that regulates awn development, grain size, and grain number in rice (Luo et al., 2013). Histological observations indicated that increased cell numbers in the longitudinal direction of spikelet hulls determined the grain length phenotype in *lgs1*. Consistent with this finding, qRT-PCR analysis showed that relative expression levels of genes involved in the cell cycle mechanisms were up-regulated in the young panicles of *lgs1*, suggesting that the increased cell numbers might result from increased expression levels of genes promoting cell proliferation. Although we found that G-box elements were located in the promoter regions of some up-regulated cell cycle genes, we obtained no evidence to show that OsbHLH107 could bind to these G-boxes and regulate expression of these cell cycle genes, directly (data not shown). Because of the suggested grain size regulator role for OsbHLH107, we examined the relative expression levels of several genes that were previously reported to act as grain size regulators. There were no significant differences in the transcript levels of these genes between WT and *lgs1* (data not shown), indicating that *OsbHLH107* may act in a new way to regulate grain size.

## Conclusions

OsbHLH107 is a member of the bHLH transcription factor family, which regulates grain size by influencing cell numbers in the longitudinal direction of spikelet hulls. We found that OsPIL11, a homolog of OsbHLH107, also regulates grain size. Our results revealed that OsbHLH107 and its homologs may play important roles in regulation of grain size in rice.

## Methods

### Plant materials and growth conditions

*lgs1* (1B-04807) was isolated from a set of activation-tagged T-DNA insertion rice lines (Jeong et al., 2002, 2006) and was kindly provided by Prof. Gynheung An (Department of Plant Systems Biotech, Kyung Hee University, Yongin, South Korea). All plants were grown in the field at two sites, Shunyi in Beijing and Sanya in Hainan province.

### Statistical analysis and histological observations

Agronomic traits were measured at the time of harvest. Harvested seeds were air-dried, and fully developed grains were measured for grain length, width, and weight. Fresh young spikelet hulls were collected and observed using an electron microscope (LEICA DM500 B). ImageJ software was used to measure the size of the inner epidermal cells. Total length of the spikelet hulls divided by the average length of the cells to obtain an approximate cell number.

### Primer design, RNA extraction, qRT-PCR, and 5’ RACE experiment

Primer Premier 5.0 software was used to design primers, and all the primers used in this study are listed in Additional file 1: Table S1. Total RNA was isolated from tissues using a RNAprep Pure Plant Kit (Tiangen Biotech Co., Ltd., Beijing). First-strand cDNA was synthesized using a PrimeScript™ II 1st Strand cDNA Synthesis Kit (TaKaRa, Dalian). Synthesized cDNAs were utilized for qRT-PCR using a SYBR *Premix Ex Taq* kit (TaKaRa) on an ABI 7500 real-time PCR system (Thermo Fisher Scientific, Waltham, MA, USA). Primers were designed using the GenScript real-time PCR (TaqMan) primer design tool (https://www.genscript.com/tools/real-time-pcr-tagman-primer-design-tool). The rice *Actin1* gene (*LOC_Os03g13170*) was used to normalize the cDNA quantity. The 5’ RACE experiment was conducted using a SMARTer RACE 5’/3’ Kit (Clontech Laboratories, Inc. Mountain View, CA, USA).

### Gene cloning, transgenic constructs, multiple sequence alignment analysis, and phylogenetic analysis

DNA and protein sequences of the genes mentioned in this study were obtained from the MSU database (http://rice.plantbiology.msu.edu/). The full-length or truncated CDS of *OsbHLH107*, *OsPIL11*, and *OsPIL14* were amplified from rice variety ‘Dongjin’ and were cloned into the pCUbi1390 vector under the control of the maize *Ubi* promoter to create overexpression constructs. Gene specific fragment of *OsbHLH107* was cloned, in sense and antisense orientations, into the LH-FAD2-1390RNAi vector (Li et al., 2013) for RNAi analysis of *OsbHLH107*. CRISPR/Cas9 technology was used to obtain knock-out mutants of *OsbHLH107*, *OsPIL11*, and *OsPIL14* (Ma et al., 2017). sgRNA sequences were obtained by using online tool (http://cbi.hzau.edu.cn/cgi-bin/CRISPR). Plasmids were introduced into ‘Dongjin’ by *Agrobacterium tumefactions*-mediated transformation (strain EHA105). Multiple sequence alignment analysis was conducted using the software DNAMAN. A phylogenic tree was constructed using the software MEGA 5.0 based on the neighbor joining method as described previously (Wang et al., 2017).

### Subcellular-localization and BiFC assays

The full-length or truncated amino acid sequences of OsbHLH107 were fused to the GFP protein under the control of the 2 × CaMV35S promoter in the transient expression vector pAN580-GFP. Rice protoplast preparation and transformation were performed as previously described (Chen et al., 2006). *OsMADS3* (*LOC_Os01g10504*) (Li et al., 2011) and *D53* (*LOC_Os11g01330*) (Zhou et al., 2013) were used as nucleus-localization markers. For BiFC assays, the CDS of *OsbHLH107* was amplified and cloned into the p2YN and p2YC vectors to form the P2YN-OsbHLH107 and P2YC-OsbHLH107 plasmids, respectively. *N. benthamiana* leaf transformation was performed as described previously (Waadt and Kudla, 2008). Fluorescence was observed using a confocal laser-scanning microscope (Zeiss LSM 780).

### Y2H assays

The CDS of *OsbHLH107* and various truncated derivatives were amplified. The PCR products were inserted into the pGBKT7 and pGADT7 vectors, and these constructs were co-transformed into yeast strain AH109 as described in the Matchmaker™ Gold Yeast Two-Hybrid System User Manual (Clontech Laboratories). The yeast liquid culture was diluted to an absorbance of 0.5 at 600 nm (A600).

## Additional file

Additional file 1: **Figure S1.** Characterization of ‘Dongjin’ (WT) and *lgs1* plants. **Figure S2.** Identification of transcripts after the T-DNA insertion site by 5’ RACE. **Figure S3.** Multiple sequence alignment of OsbHLH107 and its homologs. **Figure S4.** Relative expression analysis and molecular identification of *OsbHLH107* overexpression and CRISPR/Cas9 transgenic plants. **Figure S5.** Characterization of *OsbHLH107*-RNAi seeds on a ‘Dongjin’ (WT) background. **Figure S6. ***OsbHLH107* is broadly expressed in various tissues. **Figure S7.** Subcellular-localization of the truncated forms of OsbHLH107. **Figure S8.** Y2H assays showed that the truncated form of OsbHLH107 physically interacts with itself. **Figure S9.** Phylogenetic analysis of the reported OsbHLHs. **Figure S10.** Identification of *OsPIL11* transgenic plants. **Figure S11.** Relative expression levels of *OsPILs* in ‘Dongjin’ and *lgs1*. **Figure S12.** Statistical analysis of grain length of *OsbHLH109*-CRISPR/Cas9 (107CR) and *OsPIL11*-CRSPR/Cas9 (PIL11CR) T_1_ generation plants. **Table S1.** Primers used in this study. **Table S2.** Information regarding cell cycle genes used in this study. **Table S3.** Information regarding bHLH genes used in this study. (DOCX 5206 kb)

## Abbreviations

5’ RACE: 5’ rapid amplification of cDNA ends
aa: Amino acid
AD: Active domain
BD: Binding domain
BiFC: Bimolecular fluorescence complementation
CDS: Coding sequence
CR: CRISPR/Cas9
cv.: Cultivar
DAF: Days after fertilization
LB: Left border
nt: Nucleotide
OE: Overexpressed/Overexpression
P: Panicles
qRT-PCR: Quantitative real-time PCR
RB: Right border
RNAi: RNA interference
WT: Wild type
Y2H: Yeast two-hybrid
