# Supplementary material for: Overexpression of OsbHLH107, a member of the basic helix-loop-helix transcription factor family, enhances grain size in rice (Oryza sativa L.)
Source: Rice (N Y). 2018 Jul 20;11:41. doi: 10.1186/s12284-018-0237-y (PMC6054598; doi:10.1186/s12284-018-0237-y)
Supplement: Supplementary file 1 — Figure S1. Characterization of ‘Dongjin’ (WT) and lgs1 plants. Figure S2. Identification of transcripts after the T-DNA insertion site by 5’ RACE. Figure S3. Multiple sequence alignment of OsbHLH107 and its homologs. Figure S4. Relative expression analysis and molecular identification of OsbHLH107 overexpression and CRISPR/Cas9 transgenic plants. Figure S5. Characterization of OsbHLH107-RNAi seeds on a ‘Dongjin’ (WT) background. Figure S6. OsbHLH107 is broadly expressed in various tissues. Figure S7. Subcellular-localization of the truncated forms of OsbHLH107. Figure S8. Y2H assays showed that the truncated form of OsbHLH107 physically interacts with itself. Figure S9. Phylogenetic analysis of the reported OsbHLHs. Figure S10. Identification of OsPIL11 transgenic plants. Figure S11. Relative expression levels of OsPILs in ‘Dongjin’ and lgs1. Figure S12. Statistical analysis of grain length of OsbHLH109-CRISPR/Cas9 (107CR) and OsPIL11-CRSPR/Cas9 (PIL11CR) T1 generation plants. Table S1. Primers used in this study. Table S2. Information regarding cell cycle genes used in this study. Table S3. Information regarding bHLH genes used in this study. (DOCX 5206 kb) [file 12284_2018_237_MOESM1_ESM.docx]

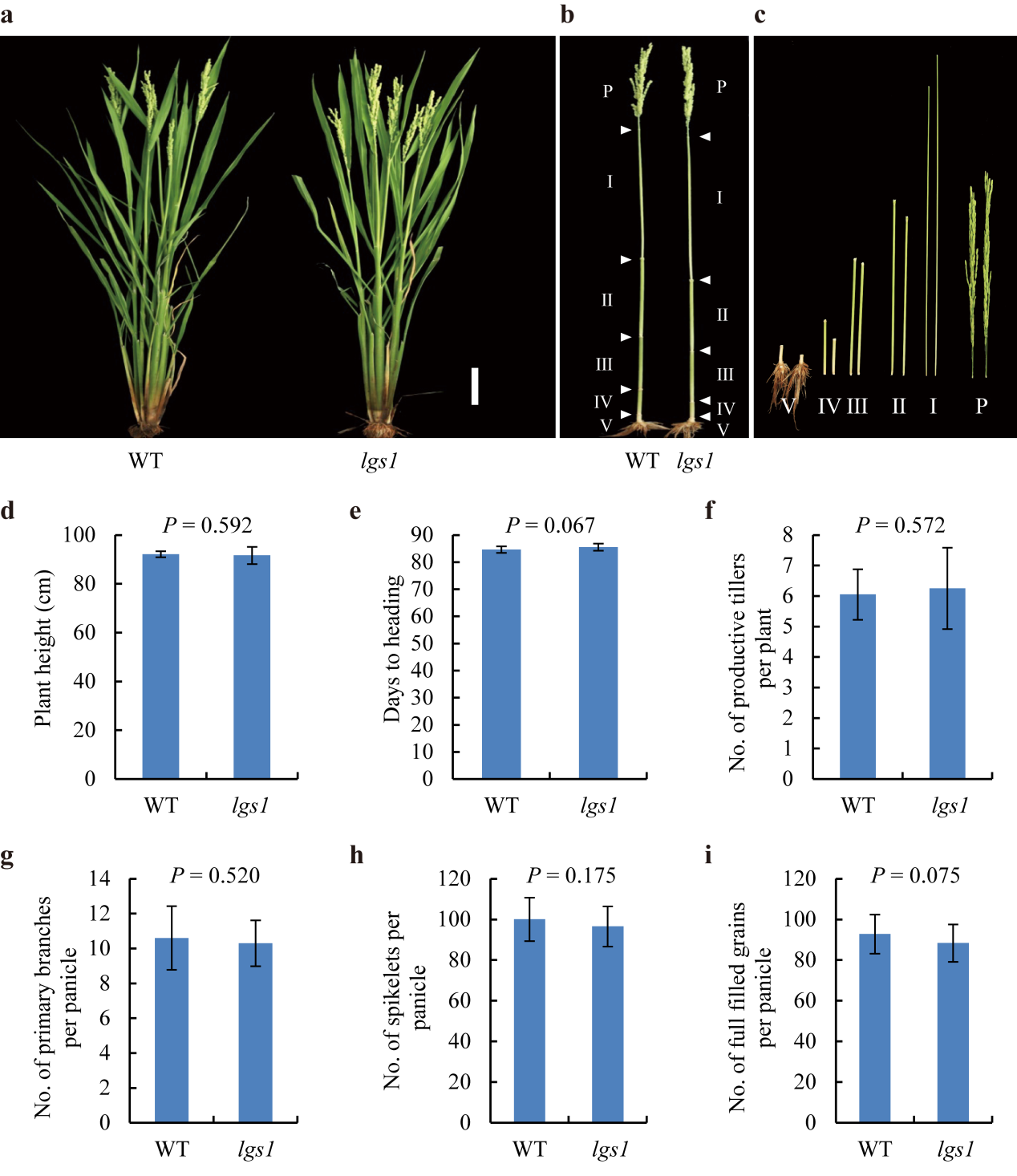


**Figure S1** Characterization of ‘Dongjin’ (WT) and *lgs1* plants. **a** Plant architecture of WT and *lgs1* at the heading stage. Scale bar, 10 cm. **b** and **c** Panicles and internodes of WT and *lgs1*. P, panicle. Internodes were labeled from I to V. White arrows in **b** indicate nodes. **d-i** Plant height (**d**, n = 20), days to heading (**e**, n = 20), tiller number (**f**, n = 20), primary branch number per panicle (**g**, n = 30), spikelet number per panicle (**h**, n = 30), and fully filled grain number per panicle (**i**, n = 30) of WT and *lgs1*. Data are the mean ± SD. Student’s *t*-tests were used to generate the *P* values.


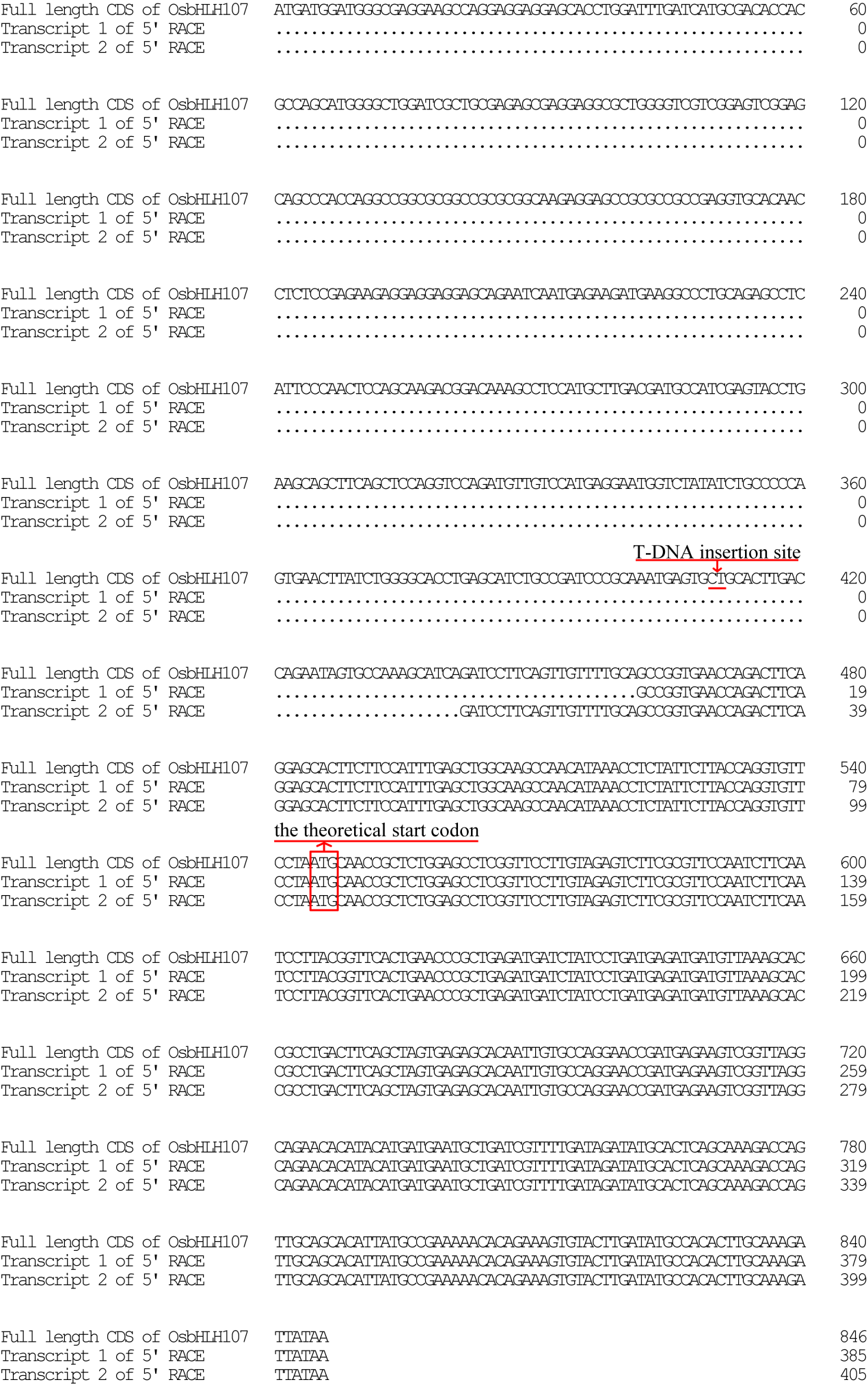


**Figure S2** Identification of transcripts after the T-DNA insertion site by 5' RACE.


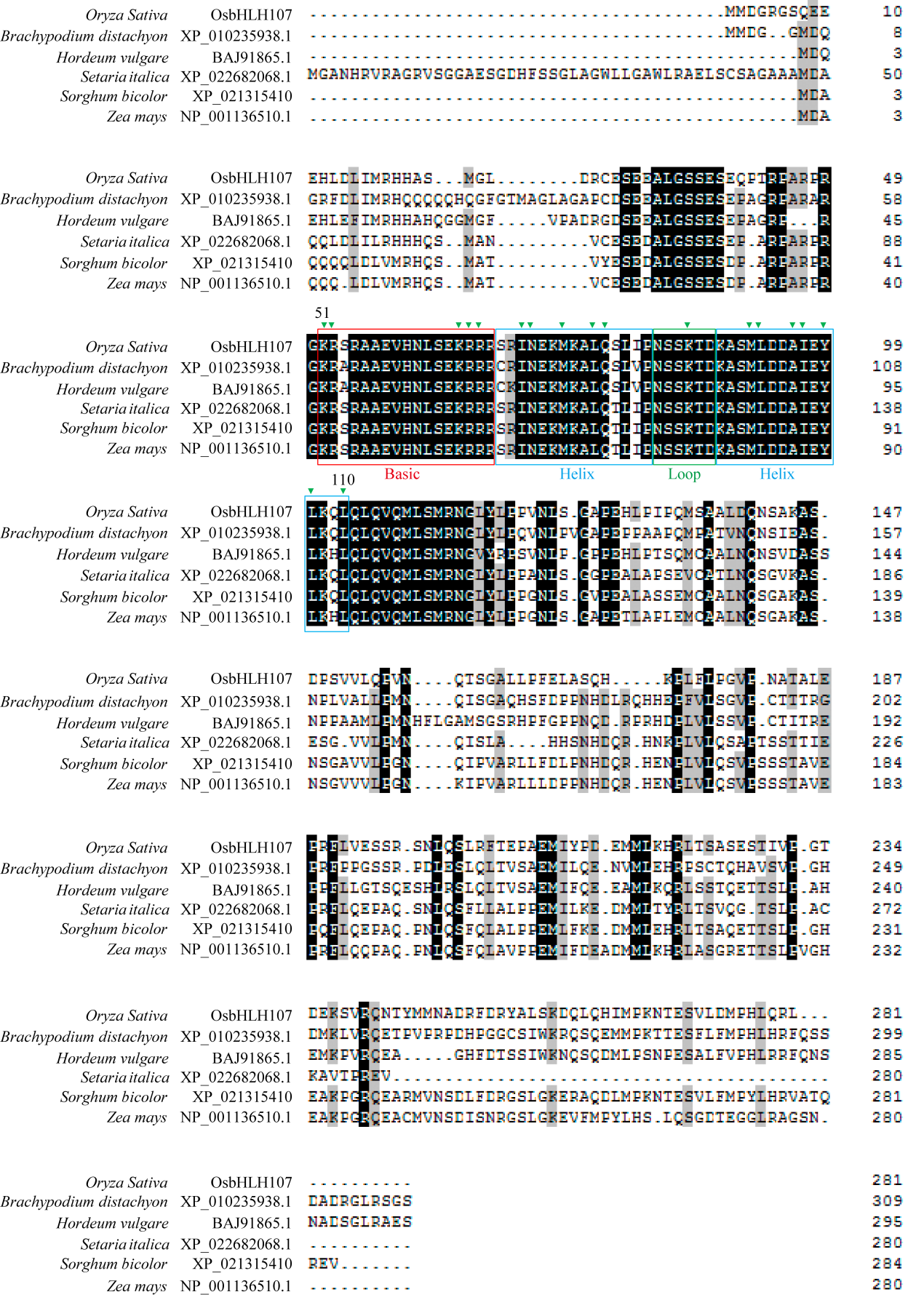


**Figure S3** Multiple sequence alignment of OsbHLH107 and its homologs. Amino acid residues identical and similar to those of OsbHLH107 are shaded in black (100%) and gray (≥75%), respectively. Numbers above sequences represent amino acid positions. The bHLH domain contains 60 amino acid residues from aa 51-110. Conserved amino acids of the bHLH domain are indicated by green arrows.


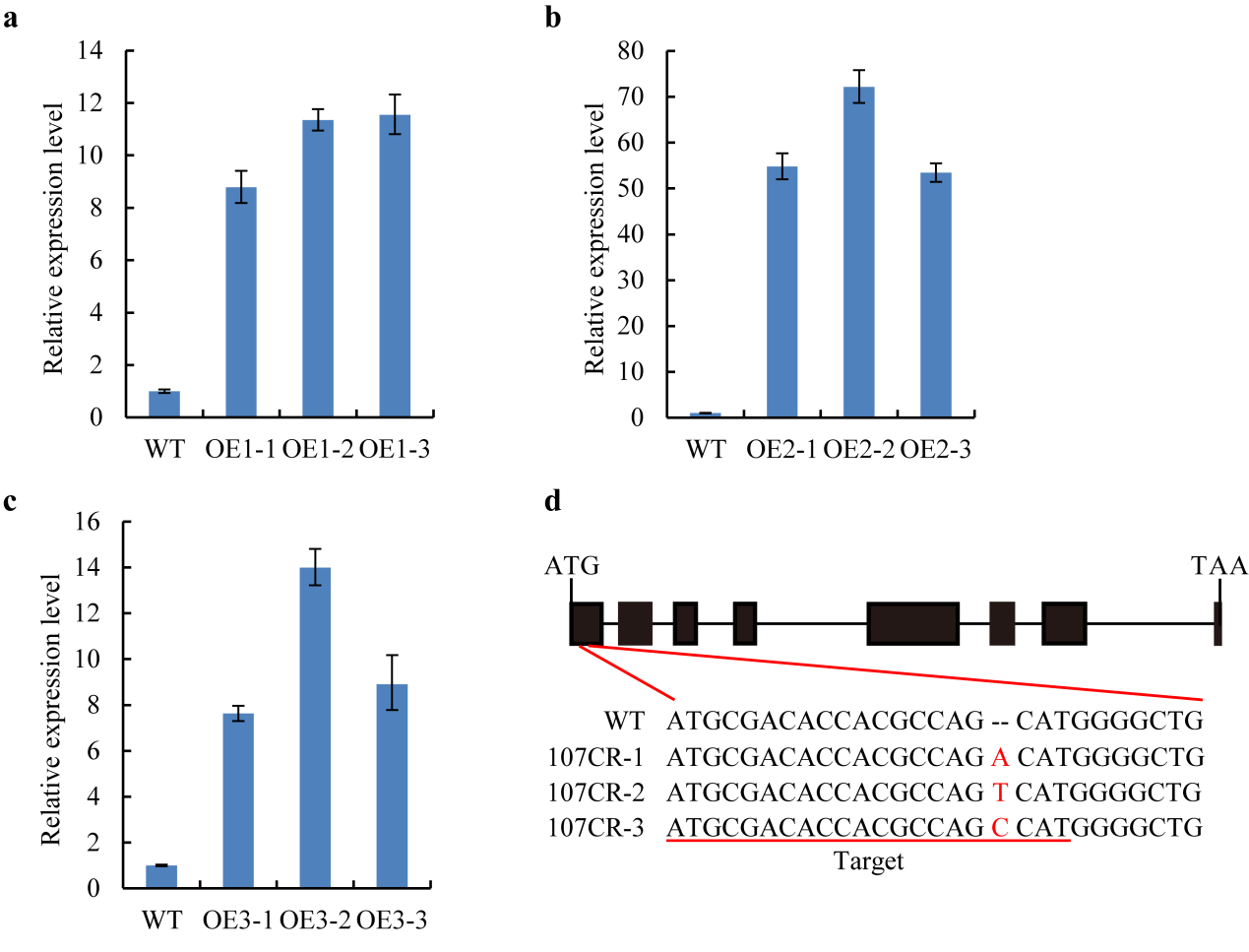


**Figure S4** Relative expression analysis and molecular identification of *OsbHLH107* overexpression and CRISPR/Cas9 transgenic plants. **a**-**c** qRT-PCR analysis of the transgenic plants is shown in **Figure 4a**-**c**, with RNA samples isolated from leaves, respectively. *OsActin1* was used as the internal control, and the values of the relative expression levels in the WT were set to one (n = 3). Data are the mean ± SD. **d** Nucleotide changes in the CRISPR/CAS9 target region of the transgenic plants are shown in **Figure 4d**.


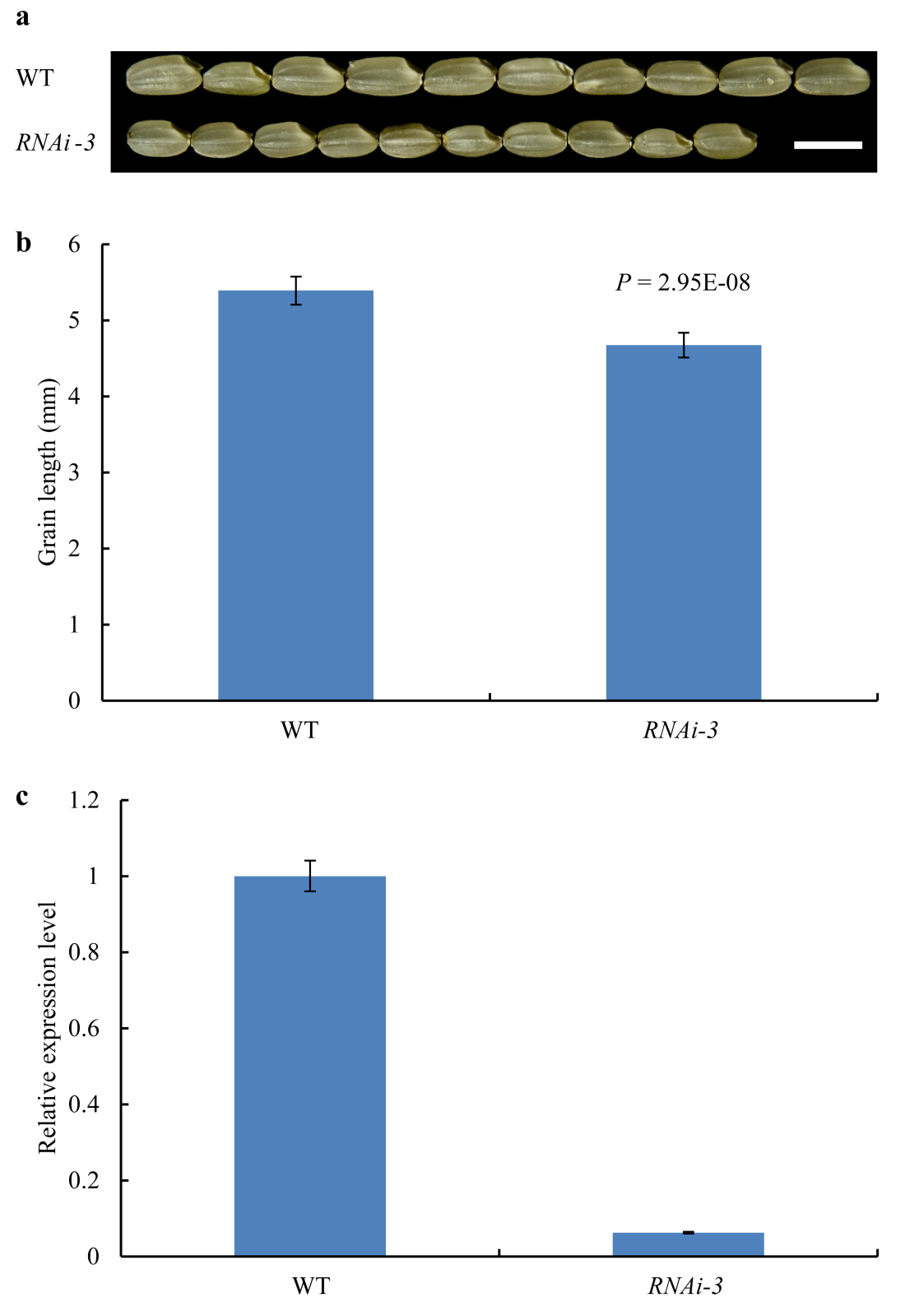


**Figure S5** Characterization of *OsbHLH107*-RNAi seeds on a ‘Dongjin’ (WT) background. (a) Grain length appearance of *OsbHLH107-*RNAi transgenic plants. Scale bar, 5 mm. (b) Statistical analysis of grain length shown in **a** (n = 10). (c) qRT-PCR analysis of *OsbHLH107*-RNAi plants with RNA isolated from leaves. *OsActin1* was used as the control, and the values of the relative expression levels in the WT were set to one (n = 3). Data are the mean ± SD. Student’s *t*-tests were used to generate the *P* values.


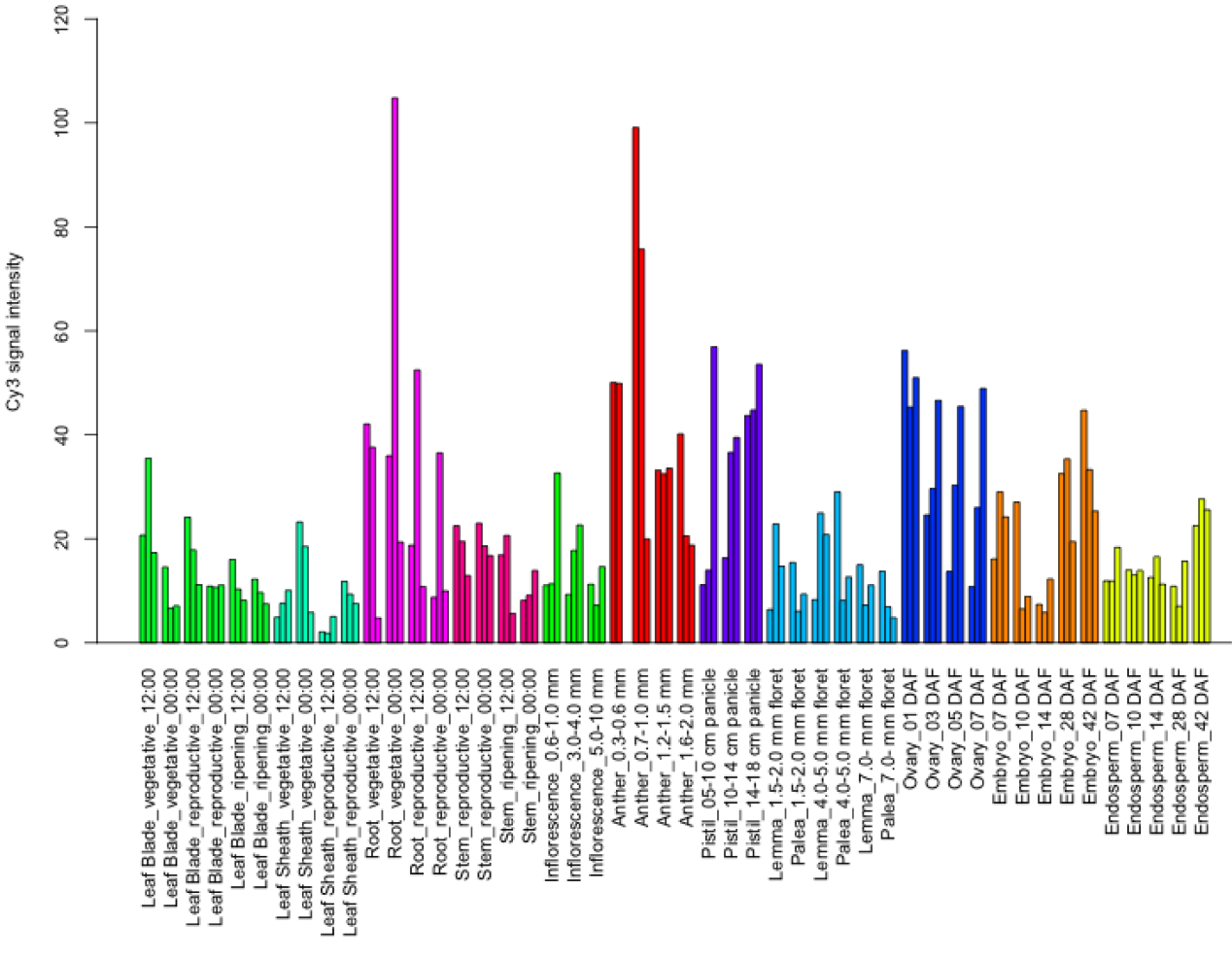


**Figure S6** *OsbHLH107* is broadly expressed in various tissues.


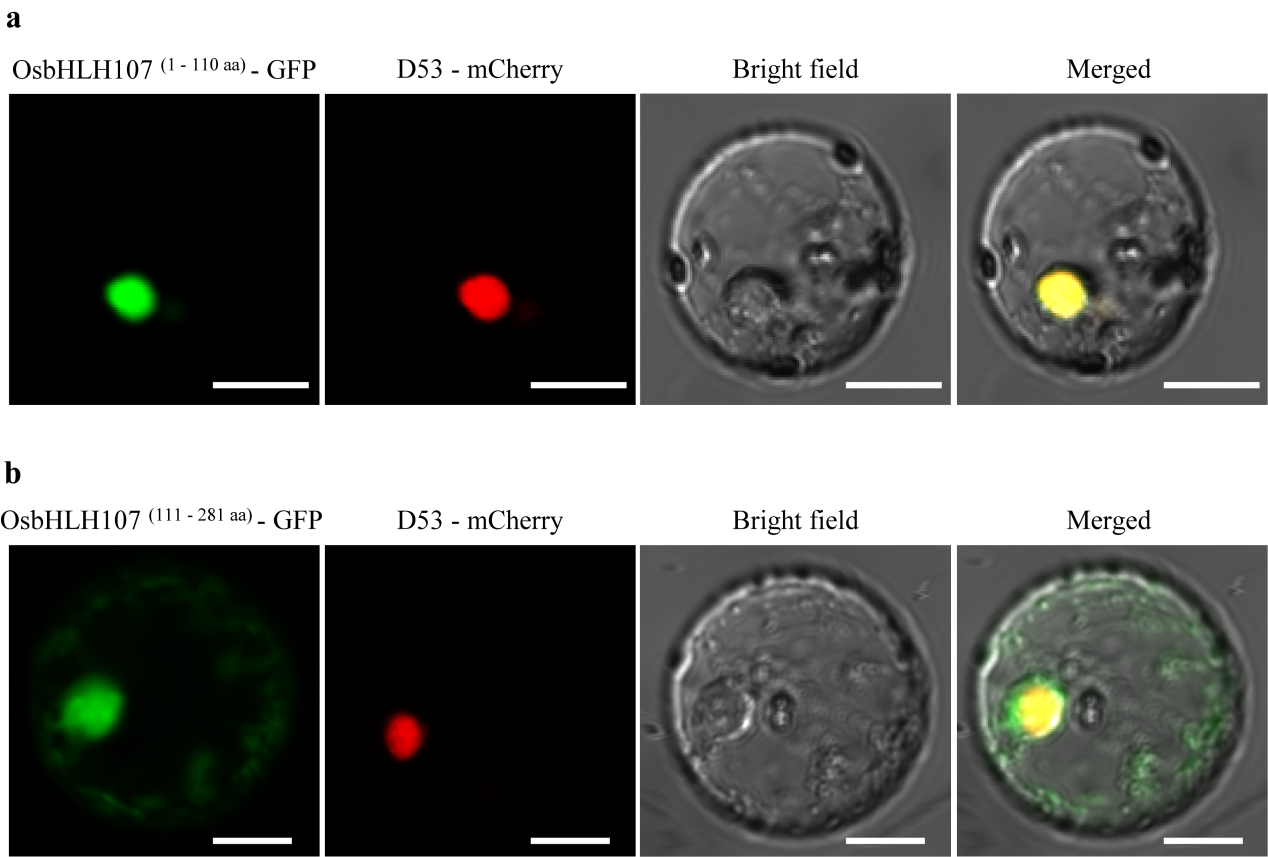


**Figure S7** Subcellular-localization of the truncated forms of OsbHLH107. Both aa 1-110 (**a**) and aa 111-281 (**b**) of the OsbHLH107 protein were located in the nucleus of rice protoplasts. *D53* was used as a nucleus-localization marker. Scale bar, 10 μm.


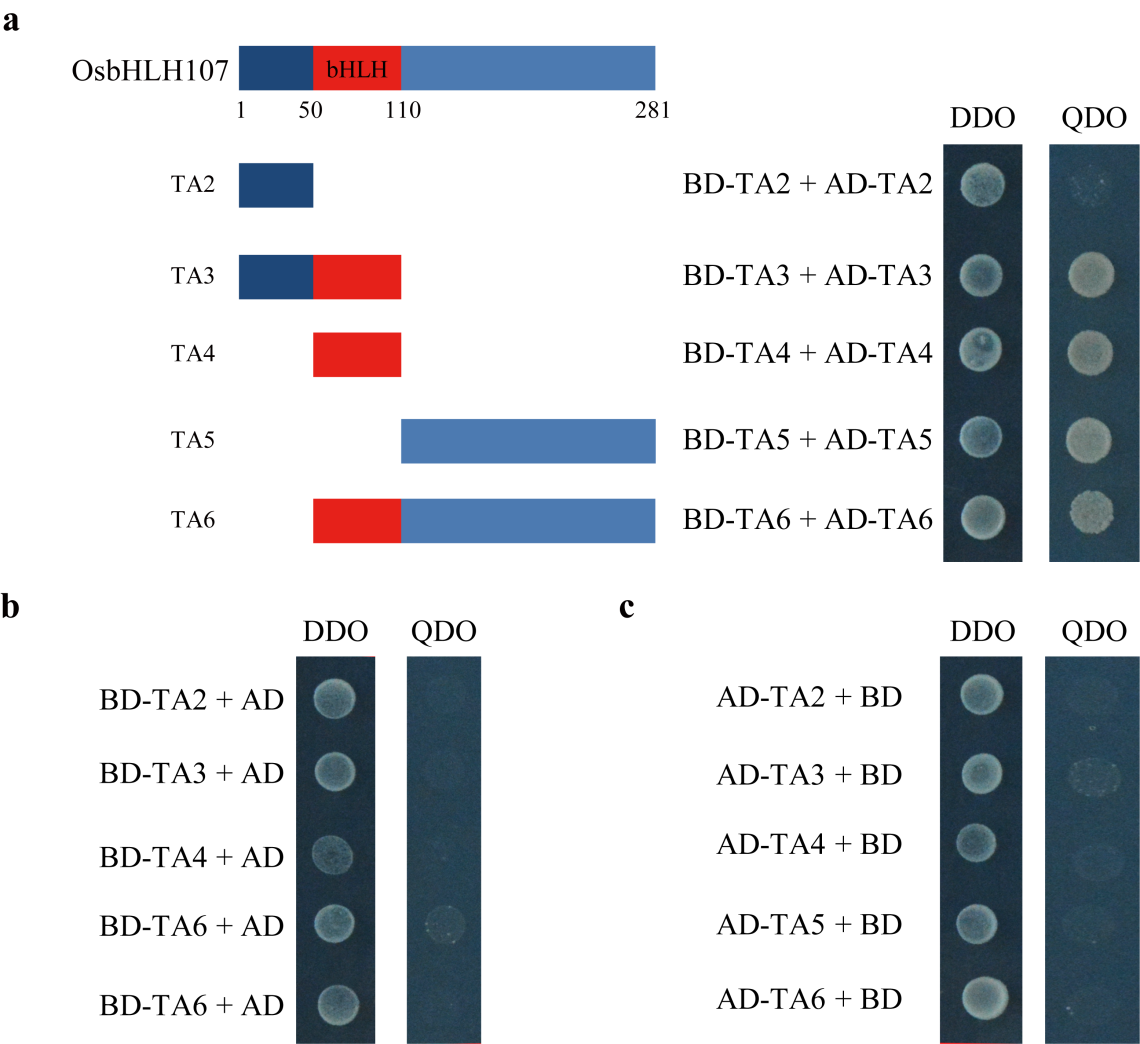


**Figure S8** Y2H assays showed that the truncated form of OsbHLH107 physically interacts with itself. AD, active domain. BD, GAL4-DNA-binding domain. DDO, SD/-Trp-Leu. QDO, SD/-Trp-Leu-Ade-His.


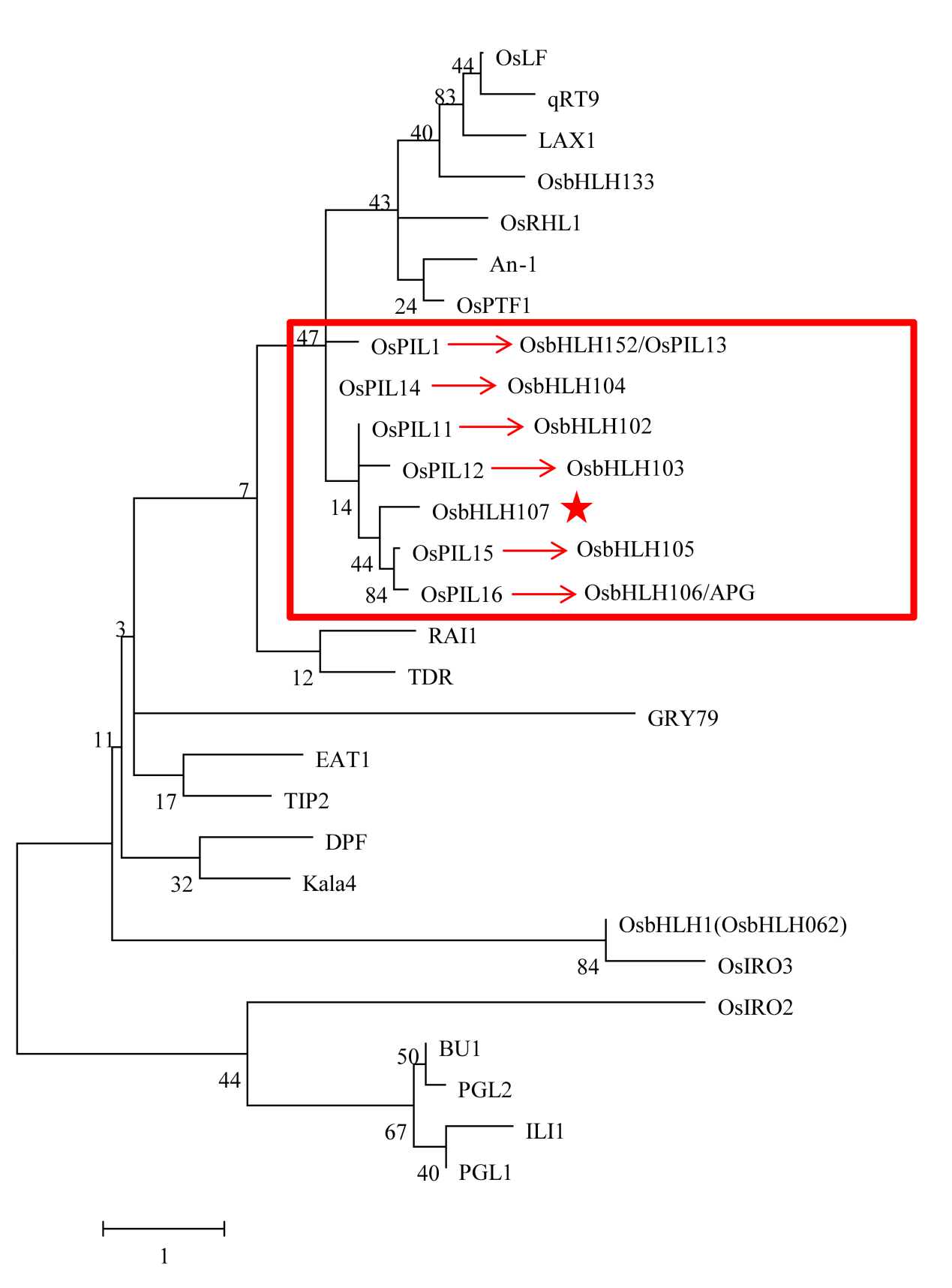


**Figure S9** Phylogenetic analysis of the reported OsbHLHs.

**
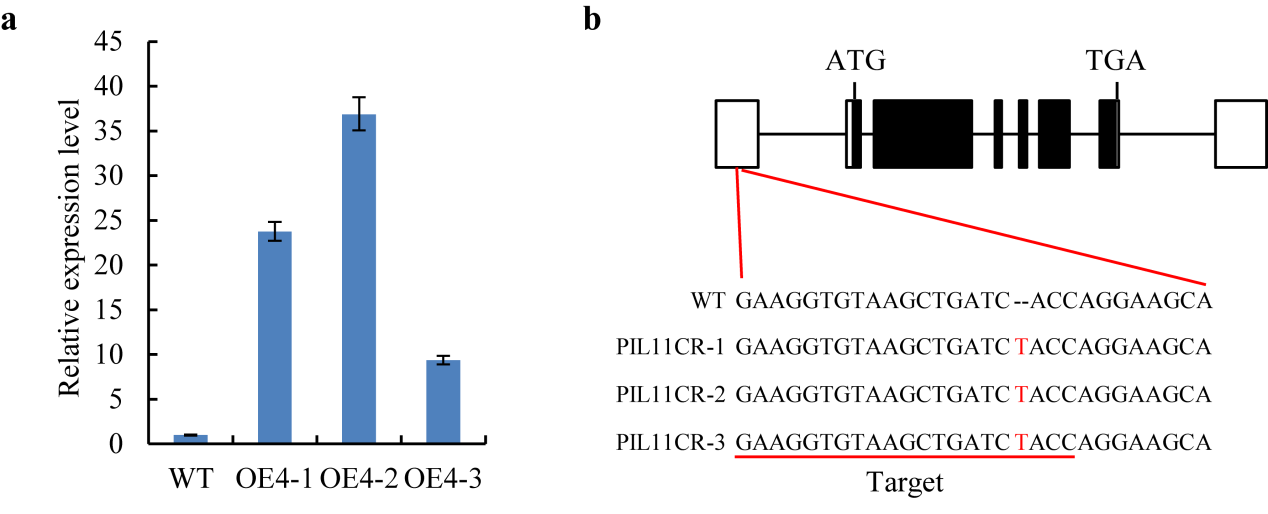
**

**Figure S10** Identification of *OsPIL11* transgenic plants. **a** qRT-PCR analysis of the transgenic plants shown in **Figure 7a** with RNA isolated from leaves. *OsActin1* was used as the control, and the values of the relative expression levels in the WT were set to one (n = 3). Data are the mean ± SD. **b** Nucleotide changes in the CRISPR/Cas9 target region of the transgenic plants shown in **Figure 7b**.


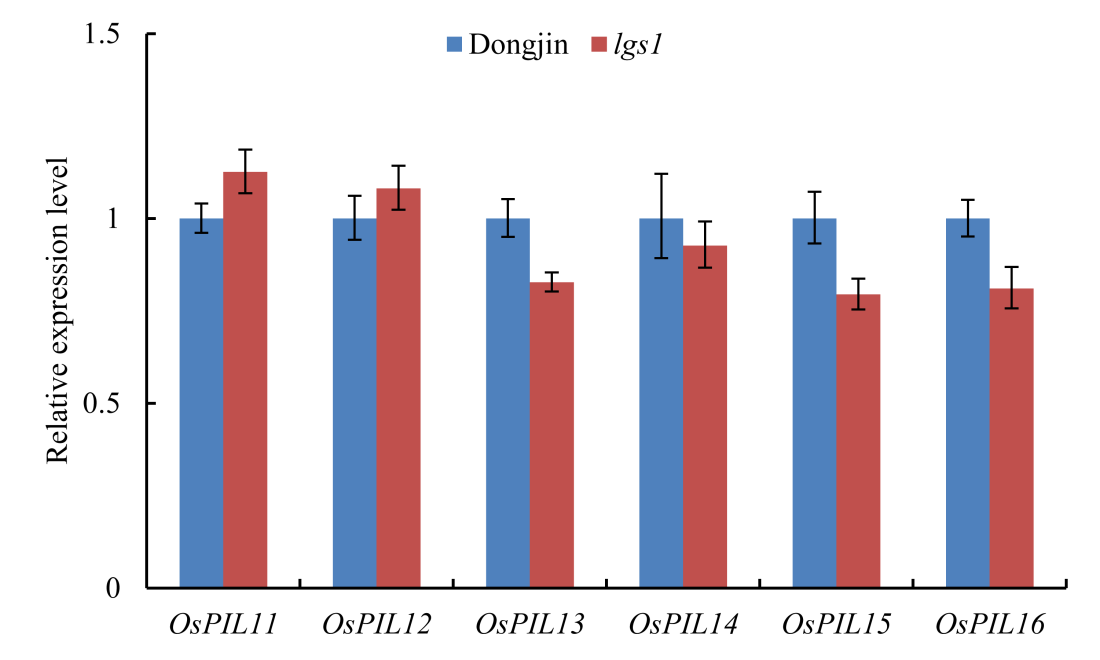


**Figure S11** Relative expression levels of *OsPILs* in ‘Dongjin’ and *lgs1*. Relative expression levels of each gene were determined by qRT-PCR with RNA isolated from leaves of ‘Dongjin’ and *lgs1*. *OsActin1* was used as the internal control, and the values of relative expression levels in ‘Dongjin’ were set to one (n = 3). Data are the mean ± SD.


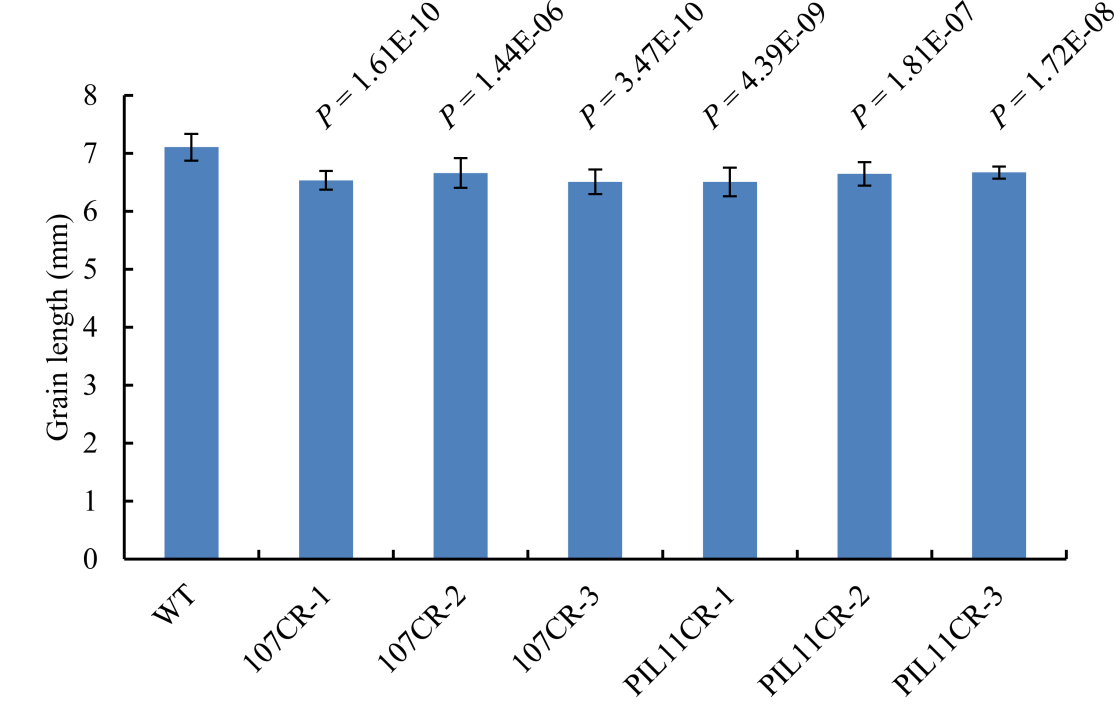


**Figure S12** Statistical analysis of grain length of *OsbHLH109*-CRISPR/Cas9 (107CR) and *OsPIL11*-CRSPR/Cas9 (PIL11CR) T_1_ generation plants. n = 20.

**Table S1** Primers used in this study.

| Purpose | Primer name | Forward primer (5'-3') | Reverse primer (5'-3') |
| --- | --- | --- | --- |
| Identification of T-DNA insertion site | P1 | ATGCCACATTGTTGTTTGC |  |
|  | P2 | TCTACAAGGAACCGAGGCT |  |
|  | P3 | CGGCGTGGTGTAGAGCATTA |  |
| qRT-PCR analysis | Qrt1 | AGGAGGAGGAGCAGAATCAA | CTGCTTCAGGTACTCGATGG |
|  | Qrt2 | ATTCCCAACTCCAGCAAGAC | ACCATTCCTCATGGACAACA |
|  | Qrt3 | AGTCTTCGCGTTCCAATCTT | AACCGACTTCTCATCGGTTC |
|  | q*LOC_Os02g56110* | CAGGCAGCTGTTCCAAAGTT | GCATTGCAAGTGGTTTCTGC |
|  | q*LOC_Os02g56120* | ATTCCCAACTCCAGCAAGAC | ACCATTCCTCATGGACAACA |
|  | q*LOC_Os02g56130* | GGAACCGGTGTCACTAACCT | ATACTCAACCACCACTGGCA |
|  | q*LOC_Os02g56150* | GAAGATCGCATACCCGAAAT | CAGCTTTGCACAGGATGATT |
|  | q*LOC_Os02g56160* | TGGAGCATCATGGACCACTC | GGCGAGGAAGTAGAGCTGAT |
|  | q*LOC_Os02g56170* | TGCTCTCGCGCTAATTGAAG | GGATCCTGATGTGGGAGGAG |
|  | qOE1 | TTCACTGAACCCGCTGAGAT | TGTGTTCTGCCTAACCGACT |
|  | qOE2 | AGTCTTCGCGTTCCAATCTT | AACCGACTTCTCATCGGTTC |
|  | qOE3 | CTCCAGCAAGACGGACAAAG | CATCTGGACCTGGAGCTGAA |
|  | qOE4 | GAGCTGCTATGGTGCAATGG | CTCGGTGAACAGGTCCTTCT |
|  | q*OsPIL11* | CGCCATTAGGCGAAGACGAC | GTCGTCGACCGGGTACTGAA |
|  | q*OsPIL12* | CCAGGACACTCGGCGAAGAT | CCGGTGGTGATTCTGGCTCT |
|  | q*OsPIL13* | CAGCAACTTCGGCGCTTCC | TCTCTTCGGTTGCCTCGCAT |
|  | q*OsPIL14* | CCAGATGCCTCAGATCCCTT | CCGCGGATGCGATTATCATT |
|  | q*OsPIL15* | GCGGACTATGGCTCCGACTT | TGTTATTGGTGGCGCCGTTG |
|  | q*OsPIL16* | TGCTGGAGGAAGCCATCGAG | TGCTGCATCTGCATGTGGTG |
|  | q*E2F2* | TGTTGGTGGCTGCCGATAT | CGCCAGGTGCACCCTTT |
|  | q*H1* | GCAAGGCACCTGCAGCTT | AGGCAGCCTTTGTACAGATCCT |
|  | q*CYCA1;1* | GTTTCGGTTGACGAGACGATGT | CGCTGCAAGGAACCTAGAACTG |
|  | q*CYCA2;1* | CTACCCGCAAGAACTGAACG | TTTGGATGAGTTGGCACAGC |
|  | q*CYCA3;2* | AGGTTGTCAAGATGGAGAGCGA | CGCTTTTTGTCTTCCTGGCA |
|  | q*CYCB1;1* | CACTCTCAAGCACCACACTGGA | ACAACCCTCAGCTTGCTCTCAG |
|  | q*CYCB2;2* | CTCAAGGCTGCACAATCTGACA | GCATTGACGGCTGGAATTTG |
|  | q*CDKB2;1* | AAGTTTGGCCAGGAGTGAGCA | TCAAGAGCATCAGCGTCGAGA |
|  | q*CYCD4;1* | GCCATGGAGTTGATACATCCAA | CCAGTAGGGCTCCGTGGAAT |
|  | q*CYCD7;1* | CCTTCCACACTGACGGTACAGTT | TGCCGCTGCCAAATAGACA |
|  | q*CDKA1* | GGTTTGGACCTTCTCTCTAAAATGC | AGAGCCTGTCTAGCTGTGATCCTT |
|  | q*CDKA2* | CGAGATTTGAAGCCCCAGAA | TCCGCGAGCTTCAATGAGTT |
|  | q*CDC20* | TCGAATCACCTGTTTGTTGGC | TGGAGACAATCCAACGCAAAG |
|  | q*CDT2* | AACCGCACCAAACACTGGAA | GCAATTCACCATCTGCACTGG |
|  | q*KN* | CACCAGCTTCAAGAGATCGTGA | CCGGAATTGAGACACAACTGC |
|  | q*MCM2* | AAGTTGGCAAAAGATCCACGG | CCCCCAAACATAGCTAGTGCAA |
|  | q*MCM3* | TTCATGCGTCACTAAATGCGAG | TGAATCTGGAAGCCCAATGTTC |
|  | q*MCM4* | CCCGAATGCGATTCTCTGAA | ACCAGTGGCATGATCAGTTGC |
|  | q*MCM5* | AAGGAGAACTGCCTGTCCATGA | AGTGGCCTTAGCTTTCACCCTC |
|  | q*MAD2* | GAGCCATGCATATTCGACGTG | GGTGTCGAAGGAATGCAGCTT |
|  | q*MAPK* | ACAGAGCAGCCGAATTTTGAGA | TTCAGCGAAGCTCACACTTGG |
|  | q*OsActin1* | TGCTATGTACGTCGCCATCCAG | AATGAGTAACCACGCTCCGTCA |
| Overexpression | OE-CDS*^OsbHLH107^* ^(nt 1-330)^ | TTACTTCTGCACTAGGTACCATGATGGATGGGCGAGGA | GAATTCCCGGGGATCCTTACAACATCTGGACCTG |
|  | OE-CDS*^OsbHLH107^* ^(nt 331-846)^ | TTACTTCTGCACTAGGTACCATGTCCATGAGGAATGGT | GAATTCCCGGGGATCCTTATAATCTTTGCAAGTG |
|  | OE-CDS*^OsbHLH107^* ^(nt 1-846)^ | CGGGGTACCCCGATGATGGATGGGCGAGGAAG | GGACTAGTCCTTATAATCTTTGCAAGTGTG |
|  | OE-*OsPIL11* | TTACTTCTGCACTAGGTACCATGAACCAGTTCGTCC | GAATTCCCGGGGATCCTCAGGAGTCAGCGGCT |
| RNAi | *OsbHLH107*-RNAi Forward | GGGGTACCCCACGGACAAAGCCTCCATGCT | CGAGCTCGCTGGCACAATTGTGCTCTCA |
|  | *OsbHLH107*-RNAi Reverse | CGGGATCCCGACGGACAAAGCCTCCATGCT | TGCACTGCAGTGCACTGGCACAATTGTGCTCTCA |
| CRISPR/CAS9 | *OsbHLH107*-CRISPR/CAS9 | AGATGATCCGTGGCAATGCGACACCACGCCAGCATGTTTTAGAGCTATGC | GCATAGCTCTAAAACATGCTGGCGTGGTGTCGCATTGCCACGGATCATCT |
|  | *OsPIL11*-CRISPR/CAS9 | AGATGATCCGTGGCAGAAGGTGTAAGCTGATCACCGTTTTAGAGCTATGC | GCATAGCTCTAAAACGGTGATCAGCTTACACCTTCTGCCACGGATCATCT |
| Subcelluar localization | OsbHLH107-GFP | GGACAGCCCAGATCAACTAGTATGATGGATGGGCG | GCCCTTGCTCACCATGGATCCTTAAGTCTGTGGAT |
|  | OsbHLH107 (1-330 nt)-GFP | CGGAGCTAGCTCTAGAATGATGGATGGGCGAG | TGCTCACCATGGATCCCAACATCTGGACCTGG |
|  | OsbHLH107 (331-846 nt)-GFP | CGGAGCTAGCTCTAGAATGTCCATGAGGAATG | TGCTCACCATGGATCCTAATCTTTGCAAGTGT |
| Transcriptional activity analysis | BD-TA1 | GAATTCATGATGGATGGGCGAGGAAG | GGATCCTAATCTTTGCAAGTGTGGCA |
|  | BD-TA2 | GAATTCATGATGGATGGGCGAGGAAG | GGATCCGCCGCGCGGCCGCGCCGGCC |
|  | BD-TA3 | GAATTCATGATGGATGGGCGAGGAAG | GGATCCCAACATCTGGACCTGGAGCT |
|  | BD-TA4 | GAATTCAAGAGGAGCCGCGCCGCCGA | GGATCCCAACATCTGGACCTGGAGCT |
|  | BD-TA5 | GAATTCTCCATGAGGAATGGTCTATA | GGATCCTAATCTTTGCAAGTGTGGCA |
|  | BD-TA6 | GAATTCAAGAGGAGCCGCGCCGCCGA | GGATCCTAATCTTTGCAAGTGTGGCA |
|  | BD-TA7 | GGATCCTCCATGAGGAATGGTCTATA | CTGCAGTAATCTTTGCAAGTGTGGCA |
| Yeast two-hybrid assay | BD-OsbHLH107 (BD-TA1) | GAATTCATGATGGATGGGCGAGGAAG | GGATCCTAATCTTTGCAAGTGTGGCA |
|  | AD-OsbHLH107 | GCCATGGAGGCCAGTGAATTCATGATGGATGGGCGAGGAAG | CAGCTCGAGCTCGATGGATCCTTATAATCTTTGCAAGTGTG |
|  | BD-TA2 | GAATTCATGATGGATGGGCGAGGAAG | GGATCCGCCGCGCGGCCGCGCCGGCC |
|  | BD-TA3 | GAATTCATGATGGATGGGCGAGGAAG | GGATCCCAACATCTGGACCTGGAGCT |
|  | BD-TA4 | GAATTCAAGAGGAGCCGCGCCGCCGA | GGATCCCAACATCTGGACCTGGAGCT |
|  | BD-TA5 | GAATTCTCCATGAGGAATGGTCTATA | GGATCCTAATCTTTGCAAGTGTGGCA |
|  | BD-TA6 | GAATTCAAGAGGAGCCGCGCCGCCGA | GGATCCTAATCTTTGCAAGTGTGGCA |
|  | AD-TA2 | GCCATGGAGGCCAGTGAATTCATGATGGATGGGCGAGGAAG | CAGCTCGAGCTCGATGGATCCGCCGCGCGGCCGCGCCGGCC |
|  | AD-TA3 | GCCATGGAGGCCAGTGAATTCATGATGGATGGGCGAGGAAG | CAGCTCGAGCTCGATGGATCCCAACATCTGGACCTGGAGCT |
|  | AD-TA4 | GCCATGGAGGCCAGTGAATTCAAGAGGAGCCGCGCCGCCGA | CAGCTCGAGCTCGATGGATCCCAACATCTGGACCTGGAGCT |
|  | AD-TA5 | GCCATGGAGGCCAGTGAATTCTCCATGAGGAATGGTCTATA | CAGCTCGAGCTCGATGGATCCTAATCTTTGCAAGTGTGGCA |
|  | AD-TA6 | GCCATGGAGGCCAGTGAATTCAAGAGGAGCCGCGCCGCCGA | CAGCTCGAGCTCGATGGATCCTAATCTTTGCAAGTGTGGCA |
| BiFC assay | P2YC-OsbHLH107 | TACGAACGATAGTTAATTAAATGATGGATGGGCGAGGAAG | CACCTCCTCCACTAGTTAATCTTTGCAAGTGTGGCA |
|  | P2YN-OsbHLH107 | TACGAACGATAGTTAATTAAATGGCGGAGTCGCTGGGACT | CACCTCCTCCACTAGTTCTATCTGCAAGGCCAACCG |

**Table S2** Information regarding cell cycle genes used in this study.

| Name in rice | Name in Arabidopsis | MSU Locus | RAP Locus |
| --- | --- | --- | --- |
| *E2F2* | *E2F2* | *LOC_Os04g18090* | *Os04g0253000* |
| *H1* | *H1* | *LOC_Os12g06200* | *Os12g0158800* |
| *CYCA1; 1* | *CYCA2; 3* | *LOC_Os01g13260* | *Os01g0233500* |
| *CYCA2; 1* | *CYCA2; 2* | *LOC_Os12g31810* | *Os12g0502300* |
| *CYCA3; 2* | *CYCA2; 1* | *LOC_Os12g39210* | *Os12g0581800* |
| *CYCB1; 1* | *CYCLa2M* | *LOC_Os01g59120* | *Os01g0805600* |
| *CYCB2; 2* | *CYCB2; 2* | *LOC_Os06g51110* | *Os06g0726800* |
| *CDKB2; 1* | *CDKB2; 1* | *LOC_Os08g40170* | *Os08g0512600* |
| *CYCD4; 1* | *CYCD4* | *LOC_Os09g29100* | *Os09g0466100* |
| *CYCD7; 1* | *CYCD3* | *LOC_Os11g47950* | *Os11g0706801* |
| *CDKA1* | *CDKA1* | *LOC_Os06g07480* | *Os06g0171700* |
| *CDKA2* | *CDKA2* | *LOC_Os02g03060* | *Os02g0123100* |
| *CDC20* | *CDC20* | *LOC_Os04g51110* | *Os04g0599800* |
| *CDT2* | *CDT2* | *LOC_Os03g49200* | *Os03g0699100* |
| *KN* | *KN* | *LOC_Os03g52650* | *Os03g0736500* |
| *MCM2* | *MCM2* | *LOC_Os11g29380* | *Os11g0484300* |
| *MCM3* | *MCM3* | *LOC_Os05g39850* | *Os05g0476200* |
| *MCM4* | *MCM4* | *LOC_Os01g36390* | *Os01g0544450* |
| *MCM5* | *MCM5* | *LOC_Os02g55410* | *Os02g0797400* |
| *MAD2* | *MAD2* | *LOC_Os04g40940* | *Os04g0486500* |
| *MAPK* | *MAPK* | *LOC_Os09g21510* | *Os09g0383300* |

**Table S3** Information of reported *OsbHLH* genes used in this study.

| Name | bHLH ID | MSU Locus | RAP-DB Locus |
| --- | --- | --- | --- |
| *OsbHLH001* | *OsbHLH001* | *LOC_Os07g43530* | *Os07g0628500* |
| *RAI1* | *OsbHLH003* | *LOC_Os03g04310* | *Os03g0135700* |
| *TDR* | *OsbHLH005* | *LOC_Os02g02820* | *Os02g0120500* |
| *DPF* | *OsbHLH025* | *LOC_Os01g09990* | *Os01g0196300* |
| *Kala4* | *OsbHLH016* | *LOC_Os04g47059* | *Os04g0557500* |
| *OsIRO2* | *OsbHLH056* | *LOC_Os01g72370* | *Os01g0952800* |
| *OsIRO3* | *OsbHLH063* | *LOC_Os03g26210* | *Os03g0379300* |
| *An-1* | *OsbHLH093* | *LOC_Os04g28280* | *Os04g0350700* |
| *OsPTF1* | *OsbHLH096* | *LOC_Os06g09370* | *Os06g0193400* |
| *OsPIL11* | *OsbHLH102* | *LOC_Os12g41650* | *Os12g0610200* |
| *OsPIL12* | *OsbHLH103* | *LOC_Os03g43810* | *Os03g0639300* |
| *OsPIL14* | *OsbHLH104* | *LOC_Os07g05010* | *Os07g0143200* |
| *OsPIL15* | *OsbHLH105* | *LOC_Os01g18290* | *Os01g0286100* |
| *OsPIL16/APG* | *OsbHLH106* | *LOC_Os05g04740* | *Os05g0139100* |
| *OsRHL1* | *OsbHLH115* | *LOC_Os06g08500* | *Os06g0184000* |
| *OsLF* | *OsbHLH119* | *LOC_Os05g46370* | *Os05g0541400* |
| *qRT9* | *OsbHLH120* | *LOC_Os09g28210* | *Os09g0455300* |
| *LAX1* | *OsbHLH123* | *LOC_Os01g61480* | *Os01g0831000* |
| *OsbHLH133* | *OsbHLH133* | *LOC_Os12g32400* | *Os12g0508500* |
| *EAT1/DTD* | *OsbHLH141* | *LOC_Os04g51070* | *Os04g0599300* |
| *TIP2* | *OsbHLH142* | *LOC_Os01g18870* | *Os01g0293100* |
| *OsbHLH148* | *OsbHLH148* | *LOC_Os03g53020* | *Os03g0741100* |
| *OsPIL1/OsPIL13* | *OsbHLH152* | *LOC_Os03g56950* | *Os03g0782500* |
| *ILI1* | *OsbHLH154* | *LOC_Os04g54900* | *Os04g0641700* |
| *PGL2* | *OsbHLH170* | *LOC_Os02g51320* | *Os02g0747900* |
| *GRY79* | None | *LOC_Os02g33610* | *Os02g0539600* |
| *PGL1/ILI6* | None | *LOC_Os03g07510* | *Os03g0171300* |
| *BU1/ILI4* | None | *LOC_Os06g12210* | *Os06g0226500* |
